# Rapamycin Modulates the Proinflammatory Memory-Like Response of Microglia Induced by BAFF

**DOI:** 10.3389/fimmu.2021.639049

**Published:** 2021-05-12

**Authors:** Jianing Wang, Chunshu Yang, Xiaoyu Hou, Jingyi Xu, Yang Yun, Ling Qin, Pingting Yang

**Affiliations:** ^1^ Department of Rheumatology and Immunology, First Affiliated Hospital, China Medical University, Shenyang, China; ^2^ Department of 1st Cancer Institute, First Affiliated Hospital, China Medical University, Shenyang, China; ^3^ Department of Nephrology, Shengjing Hospital of China Medical University, Shenyang, China; ^4^ Department of Physiology, College of Basic Medical Science, China Medical University, Shenyang, China

**Keywords:** B cell-activating factor, microglia, trained immunity, aerobic glycolysis, mammalian target of rapamycin

## Abstract

**Background:**

Recently trained immunity of microglia provided an opportunity to study the chronic effect of microglial activation and its metabolic rewiring in neuroimmunological diseases. Since elevated levels of B cell-activating factor (BAFF) have been proved to be associated with some chronic neuroimmunological disorders. Here, we used the trained innate immunity model to analyze the effect of BAFF, a vital regulator of the adaptive immune system, on long-term microglial activation and metabolic reprogramming *in vitro* and *in vivo*.

**Methods and results:**

In vitro, BV2 cells and mouse primary microglial cells were incubated with BAFF for 24 h (BAFF priming). After 5 days of resting, microglia were restimulated with LPS (LPS restimulation) or BAFF (BAFF restimulation). BAFF priming induced a pro-inflammatory trained immunity-phenotype of both BV2 cells and primary microglial cells, which was indicated by morphological change, secretion of pro-inflammatory cytokine and chemokine upon LPS restimulation or BAFF restimulation. The production of lactate and NAD+/NADH ratio were elevated 5 days after BAFF priming. The activation of the Akt/mTOR/HIF-1α pathway was induced by BAFF priming and lasted for 5 days. Pretreating the BV2 cells or mouse primary microglial cells with rapamycin blocked mTOR/HIF-1α activation and cellular metabolic reprogramming induced by BAFF training. Consistently, rapamycin efficiently suppressed the trained immunity-like responses of microglia triggered by BAFF. In vivo, adult male mice were treated with BAFF by intracerebroventricular injection for priming and 7 days later with BAFF for restimulation. BAFF training activated microglia in the cortex and hippocampus. The production of proinflammatory cytokines and chemokines was elevated after BAFF training.

**Conclusion:**

Our current data, for the first time, demonstrate that BAFF priming induces a proinflammatory memory-like response of microglia not only to LPS but also to BAFF itself. Rapamycin inhibits microglial priming triggered by BAFF through targeting the mTOR/HIF-1α signaling pathway. Our data reveal a novel role of BAFF in trained immunity and that rapamycin may be a potential therapeutic target of neuroimmunological diseases.

## Introduction

Trained immunity, also termed innate immunity memory, has been proposed as a mechanism for innate immune cells to remember an inflammatory stimulus and respond in an enhanced manner to future exposure to the same or an unrelated pathogen (1). The resulting enhanced response upon a secondary challenge is characterized by increased cytokine production and relevant metabolic and epigenetic changes. Other than pathogens, metabolites or inflammatory stimuli such as oxidized low-density lipoprotein (oxLDL) or Liver X Receptor (LXR) can trigger trained immunity phenotype of monocytes which plays an essential role in human atherosclerosis (2, 3). However, monocytes in periphery circulation are short-lived, and maintenance of innate memory mainly depends on the update of circulating precursors. In contrast to circulating monocytes, most tissue-resident macrophages possess a longer life span and maintain themselves independently of circulating precursors. Microglia persist in the central nervous system (CNS) throughout adulthood and is the most long-lived tissue-resident macrophage (4). In this term, immune memory of microglia in the immune-privileged brain would potentially affect the severity of any neurological diseases involving an inflammatory component. Recent studies have brought microglial trained immunity into focus. Wendeln et al. confirmed that microglia could be prepared to confer long-lasting memory (5). By peripherally (i.p.) administering lipopolysaccharide (LPS), microglia developed a proinflammatory memory. Moreover, peripheral immune stimulation amplifies microglia immune responses months later and aggravates disease burden in mouse models of neurological diseases, including Alzheimer’s disease (AD) and strokes. These functional and phenotypic changes of microglia sustained even after the initial trigger had subsided. Other than LPS, individual cytokines such as TNF-α and IL-1β also triggered innate memory effects in the brain (5, 6), suggesting various immune stimuli may elicit immune memory of microglia. With these regards, it will be necessary to uncover whether other incentives would lead to long-term modulation of microglial responses and thereby contribute to the progression of many neuroimmunological diseases.

B cell-activating factor (BAFF) belongs to the tumor necrosis factor (TNF) family and plays a well-defined role in the adaptive immune system. BAFF is originally expressed as a full-length 285-amino acid transmembrane protein and can be released as a soluble ligand following processing by a furin-like protease (7, 8). The soluble BAFF (sBAFF) is thought to be a functional form *in vivo* and is produced by monocytes, macrophages, dendritic cells, activated T cells, and malignant B cells (9–11). BAFF binds to three classes of receptors, including B-cell maturation antigen (BCMA), transmembrane activator and calcium modulator and cyclophilin ligand interactor (TACI), and canonical BAFF receptor (BAFF-R) (9). Upon binding to its receptors, BAFF plays a vital role in the survival of B lymphocytes and ensures effective humoral immune responses (12–14).

In addition to its involvement in the adaptive immune system, BAFF has been revealed to promote pro-inflammatory responses of innate immune cells, including monocytes, macrophages, and microglia (15–19). sBAFF was found elevated in the circulation and cerebral spinal fluid (CSF) in neuroimmunological diseases, including multiple sclerosis (MS) (20), neuropsychiatric systemic lupus erythematosus (NPSLE) (21), brain Behcet’s disease (BD) (22), and optic neuritis (23). Jim et al. confirmed the proinflammatory effect of BAFF on microglial activation *in vitro* (19). BAFF displayed a synergistic effect together with SLE serum on microglial M1 activation *in vitro* (18). Emerging pieces of evidence demonstrate that BAFF plays a regulatory function in the innate immunity system, especially in microglial activation. Chronic BAFF stimulation primes B cells to reprogram to the glycolytic metabolism, which is essential for antibody production (24), suggesting a role of BAFF in the cross-talk of immune response and metabolic reprogramming. However, the effect of the cerebral elevated BAFF on long-term microglial activation and microglial cellular metabolism is still unknown.

For these reasons, we take advantage of the trained immunity model to study the chronic effect of excess BAFF on microglial activation and metabolism *in vitro* and *in vivo*. In the current study, microglia were pre-exposed with BAFF, and the inflammatory responses to restimulation of BAFF or LPS were investigated *in vitro*. In vivo, the adult mice were treated with either BAFF (BAFF priming) or artificial cerebrospinal fluid by intracerebroventricular injection and 7 days later with BAFF restimulation. Microglia activation *in vivo* was evaluated by Iba-1 staining in the cortex and hippocampus of mice. To our knowledge, this is the first study to show the influence of BAFF on microglial trained immunity-like phenotype both *in vitro* and *in vivo*. Furthermore, the current study highlights the immense impact of BAFF on microglial trained immunity and brings the therapeutic target rapamycin into the spotlights.

## Materials and Methods

### Reagents

Dulbecco’s Modified Eagle Medium (DMEM)/high glucose, DMEM/F12 (1:1), penicillin, and streptomycin were bought from Hyclone (Logan, UT, USA). Fetal bovine serum (FBS) was bought from Clark (USA). Recombinant Mouse BAFF Protein (cat 8876-BF) was purchased from R&D Systems (Minneapolis, MN, USA). Rapamycin (cat 10997), 2-Deoxy-d-glucose (2-DG, cat 13966), and 2-Methoxyestradiol (2-ME, HY-12033) were purchased from MedChemExpress (MCE) (New Jersey, USA). MK2206 (S1078) was purchased from Selleck Chemicals (USA). MTT, dimethyl sulfoxide (DMSO), and Trypan blue were bought from Sigma (St. Louis, MO, USA). Interleukin (IL)-6 (EK206/3), TNF-α (EK0527), IL-1β (EK201B/3), CCL2 (EK287/2) ELISA kits were obtained from MultiSciences (LIANKE) Biotech, Co., Ltd (Hangzhou, China). Lactate Colorimetric Assay Kit (KGT023) was purchased from Keygene Biotech (Nanjing, China). Fluorimetric Nicotinamide adenine dinucleotide (NAD+)/reduced form of NAD+(NADH) Ratio Assay Kit (cat 15263) was purchased from AAT Bioquest, Inc (CA, US). RIPA buffer, phenylmethylsulfonyl fluoride (PMSF), BCA kit, CHIP assay kit (P2078), DNA purification kit (D0033), and Rabbit polyclonal IgG (A7016) were purchased from Beyotime (Jiangsu, China). Polyvinylidene fluoride (PVDF) microporous membrane was bought from Millipore (Bedford, MA, USA). Phospho-mammalian target of rapamycin (mTOR) (Ser2448) antibody (2971S), phospho-Akt (Ser473) antibody (9271S), Hypoxia-inducible factor 1 alpha (HIF-1α) (D1S7W) rabbit monoantibody (36169S) and H3k4me3 rabbit polyantibody (9727S) were obtained from Cell Signaling Technology (Danvers, MA). Ionized calcium-binding adaptor molecule 1 (Iba1) antibody (ab153696) was purchased from Abcam (USA). mTOR antibody (20657-1-AP) and Akt antibody (10176-2-AP), HRP-conjugated Goat Anti-Rabbit IgG (H+L) (SA00001-2), and HRP-conjugated Goat Anti-Mouse IgG (H+L) (SA00001-1) were purchased from Proteintech Group, Inc (Wuhan, China). Tubulin antibody (TA-10) was bought from ZSGB bio (Nanjing, China). PE-labeled anti-BCMA, anti-TACI, and anti-BAFF-R antibodies used for flow cytometry were purchased from Miltenyi Biotec (Bergisch Gladbach, Germany). Axygen^®^ AxyPrep™ Multisource RNA Miniprep Kit was bought from Axygen Biosciences (Union City, CA, USA). The First Strand complementary DNA (cDNA) Synthesis Kit, SYBR green two-step qRT-PCR kit was bought from TAKARA (Dalian, China). Agarose, 4S Red Plus Nucleic Acid staining, siRNAs, and primers used in the PCR were purchased from Sangon Biotech (Shanghai, China). Paraformaldehyde, sucrose, 10% donkey serum, and DAPI used in the immunohistochemistry were bought from Solarbio (Beijing, China).

### BV2 Microglial Cell Line Cultures

The immortalized mouse microglial cell line (BV2) was originally obtained from the Cell Resource Centre, Peking Union Medical College. BV2 cells were cultured in DMEM/high glucose. 24 h after plated at a density of 35,000 cells/ml, BV2 cells were primed with different doses of mouse recombinant BAFF (“BAFF priming”) for 24 h. Cells were pre-incubated for at least an hour with 1 mM 2-Deoxy-d-glucose (2-DG) or 100 nM rapamycin before priming to block glycolysis or mTOR, respectively. The medium was changed after 24 h and cells were rested for 5 days. On day 6, cells were re-stimulated with 100 ng/ml LPS (“LPS restimulation”) or 40 ng/ml BAFF (“BAFF restimulation”). All the compounds were dissolved in 0.1% DMSO in DMEM/high glucose because 2-DG and rapamycin were dissolved in DMSO. The control group is 0.1% DMSO in DMEM/high glucose and described as “CTL.” The cells were incubated in a 5% CO2 incubator maintained at 37°C. 24 h after the last stimulation, cellular supernatants or cell lysates were harvested.

### Primary Mouse Microglial Cell Culture

Postnatal (P1) C57BL/6 mice pups were used in primary microglial cell culture. In brief, the brains were aseptically removed. The cerebral cortex was dissected in D’HANKS buffer and then digested with 0.25% trypsin and 0.01% DNase type I at 37°C for 8 to 10 min. The digestion was stopped by DMEM/F12 (1:1) containing 10% FBS. Cells were then collected by centrifugation at 1,000 rpm for 10 min, resuspended with DMEM/F12 (1:1) containing 10% FBS and 100 units/ml of penicillin and 100 µg/ml of streptomycin and then plated in 25 cm^2^ culture flasks for 2 h to remove the fibrocytes. Then the floating cells were removed to 75 cm^2^ culture flasks. The cells were incubated at a 37°C incubator with 5% CO2 and 95% humidified air to form confluent mixed glial layers. 7 to 10 days later, microglia growing at the top layer were collected by digesting with 0.75% trypsin for about 10 to 20 min. Cells were then centrifugated at 1,000 rpm for 10 min, and cell pellets were resuspended with DMEM/F12 (1:1) containing 10% FBS and 100 units/ml of penicillin and 100 µg/ml of streptomycin. Harvested cells were then seeded to 96 well-plate (10,000 cells/well) or 24-well plate (25,000 cells/well) pre-coated with poly-L-lysine for experiments.

### Morphology

Microglial morphology was assessed by phase-contrast microscopy based on previously described criteria (25). The cell morphology was observed using the Nexcope NIB410 microscope linked to the DLAIPU PUBLIC software (China) for image processing. Representative bright-field images were obtained using a 40× objective lens.

### Cell Viability and Cell Proliferation

Cell viability was determined using the MTT [3-(4,5-dimethylthiazol-2-yl)-2,5-diphenyltetrazolium bromide] and Trypan blue exclusive assay. Briefly, BV2 cells were seeded into 96-well plates at a density of 5,000 cells/well. 24 h after the last stimulation, 25 μl of MTT dye (5 mg/ml) was added to each well. The cells were incubated for 4 h at 37°C. Each well was then aspirated, and 150 µl DMSO was added. The optical density (OD) was determined at 490 nm in the Bio-Tex ELx808 Microplate Reader (Bio-Tex Instruments, Inc, Winooski, VT, USA). The cell viability was represented as the OD ratio of each group to the control group and was expressed as the mean OD ratio ± SEM.

The trypan blue exclusion assay was performed as described previously (26). Similar to the MTT assay, the cells were stained with 0.4% trypan blue dye and then counted using a hemocytometer. Microglial proliferation was based on total cell counts of five randomly selected fields per culture well. Values were normalized to control wells (medium only) to get fold change in proliferation.

### Cytokine and Chemokine Assay

Supernatants of microglia cultured under different conditions were collected from several experiments and centrifuged at 300*g* for 10 min at 4°C to remove cell debris. The supernatants were stored in aliquots at −80°C before use. IL-6, TNF-α, IL-1β, C-C motif ligand (CCL) 2 were measured in the culture supernatants by enzyme-linked immunosorbent assay (ELISA) according to the manufacturer’s instructions. The OD was determined at 450 nm in the Bio-Tex ELx808 Microplate Reader (Bio-Tex Instruments, Inc, Winooski, VT, USA), and the corresponding mouse recombinant protein was used as standard. All treatments were completed at least three times, and the data were expressed as the mean pg/ml ± SEM.

### Metabolite Measurements

Culture medium was collected on day 1 and day 6. The lactate concentrations in the supernatants were determined by Lactate Colorimetric Assay Kit according to the manufacturer’s instruction. NAD+ and NADH concentration was determined by Fluorimetric NAD+/NADH Ratio Assay Kit (cat15263) from the cell lysate according to the manufacturer’s protocol. All the metabolite measurement data presented were from at least three experiments.

### Western Blot

Cellular proteins were extracted from the BV2 or primary microglia cells using RIPA buffer with 1 mM PMSF. According to the manufacturer’s instructions, the lysate supernatant’s protein concentration was measured using a BCA kit. Proteins (20–40 µg) in the cell extracts were denatured with SDS sample buffer and separated using 10% SDS-polyacrylamide gel electrophoresis (PAGE). The proteins were transferred to a PVDF microporous membrane and blocked with 5% bovine serum albumin (BSA) for 2 h at room temperature. The membrane was incubated with the primary antibody overnight at 4°C. The following primary antibodies were used: Phospho-mTOR (Ser2448) antibody (1:1,000, CST) and phospho-Akt (Ser473) antibody (1:1000, CST), mTOR antibody (1:300, Proteintech), and Akt antibody (1:400, Proteintech), HIF-1α antibody (1:1,000, CST), Tubulin antibody (1:1,000, ZSGB bio). After adding the HRP-conjugated goat anti-rabbit or anti-mouse secondary antibody (1:5,000, Proteintech) and incubating for 1 h, the protein bands on the membranes were detected with a high-sig chemiluminescence Western blotting substrate (Tanon, Shanghai China), and the images were captured with the Tannon 5200Multi Imager (Tannon, Shanghai, China). Densitometric analysis of the bands was performed using the ImageJ software (NIH, Bethesda, MD, USA). The data were quantified as the relative intensity normalized to the intensity of Tubulin ± SEM.

### Flow Cytometry

The BV2 cells were stained with PE-labeled anti-BCMA, anti-TACI, and anti-BAFF-R antibodies and incubated for 15 to 20 min. The relevant isotype control antibodies were used to evaluate the background. Analysis of the cells was performed using a BD FACScan flow cytometer (Becton, Dickinson and Company, New Jersey, USA) and FlowJo software (version 7.6.1, Tree Star, Ashland, OR).

### Real-Time Polymerase Chain Reaction (qPCR) Analyses

The total RNA was extracted from BV2 cells using Axygen^®^ AxyPrep™ Multisource RNA Miniprep Kit following the manufacturer’s instruction, and the RNA was reverse-transcribed into cDNA using First Strand cDNA Synthesis Kit. Standardization was performed with GAPDH as the endogenous control. The real-time PCR was performed with the SYBR green two-step qRT-PCR kit. Real-time PCR was analyzed with the TAKARA Thermal Cycler Dice™ Real time system (TAKARA, Dalian, China). mRNA levels were determined using the 2^−δδCt^ method. Results are expressed as the fold changes of mRNA in untreated cells (CTL).

The specific primer were as follows: for TACI forward (F) 5-GTGTGGCCACTTCTGTGAGA-3 and reverse (R) 5-CTGGTGCCTTCCTGAGTTGT-3; BAFFR forward (F) 5-GTGCCTTCAGATGGTTGGAT-3 and reverse (R) 5-CCATACCTCCAGCCCAGTAA-3; BCMA forward (F) 5-ATCTTCTTGGGGCTGACCTT-3 and reverse (R) 5-CTTTGAGGCTGGTCCTTCAG-3; GAPDH forward (F) 5-CATGGCCTTCCGCGTTCCTA-3 and reverse (R) 5-ATGCCTGCTTCACCACCTTCT-3.

### Transfection

**Figure 1:**
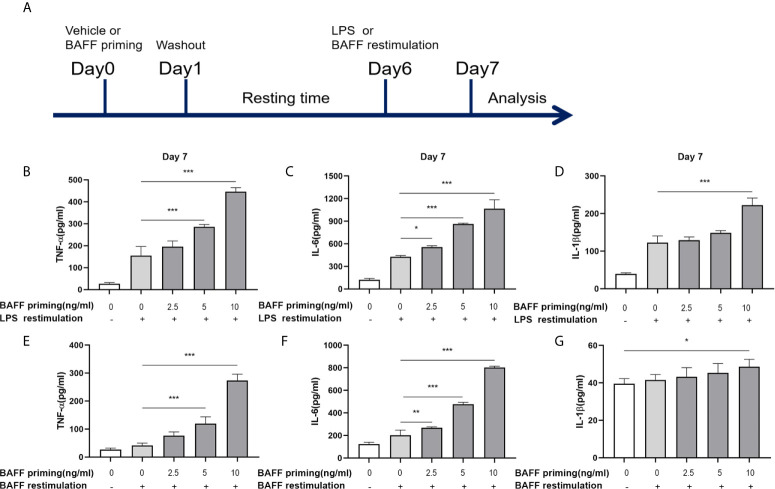
BAFF priming induced a proinflammatory memory response of BV2 cells to LPS and BAFF itself. **(A)** Graphical outline of the *in vitro* methods. BV2 cells or primary mouse microglial cells were incubated for 24 h with vehicle or BAFF (defined as ‘BAFF priming’), and after a 5-day rest period, microglial cells were restimulated with 40 ng/ml BAFF (BAFF restimulation) or 100 ng/ml LPS (LPS restimulation) for 24 h. In **(B–D)**, BV2 cells were primed with different doses of BAFF (2.5, 5, 10 ng/ml) or vehicle for 24 h, rested for 5 days, on day 6 treated with LPS restimulation for 24 h. TNF-α **(B)**, IL-6 **(C)**, and IL-1β **(D)** were measured in the supernatants by ELISA on day 7(n = 12). On day 6, BV2 cells were restimulated with BAFF instead of LPS. On day 7, TNF-α **(E)**, IL-6 **(F)** and IL-1β **(G)** were measured in the supernatants (n = 12). Data are means ± SED; Kruskal-Wallis test with Turkey *post hoc* test; *p < 0.05, **p < 0.01, and ***p < 0.001.

TACI small interfering RNA (siRNA) (Sangon Biotech, China) or control siRNA (Sangon Biotech, China) was transfected into BV2 cells (2.5106 cells) with the use of Lipofectamine™ 3000 (Invitrogen, USA). After 16-h incubation, cells were subjected to experiments for the BAFF training procedure, as shown in **Figure 1A**.

### Chromatin Immunoprecipitation Assays

According to the manufacturer’s instructions, primed cells were harvested on day 6 for Chromatin Immunoprecipitation (ChIP) assays using the CHIP assay kit (Beyotime, China, P2078). The chromatin of 2×10^6^ cells was immunoprecipitated with an antibody raised against H3k4me3 (CST, #9727) at 4°C overnight. Rabbit polyclonal IgG (Beyotime, A7016) was used as a negative control. Immunoprecipitated DNA was harvested with a DNA purification kit (Beyotime, D0033). Quantitative PCR of immunoprecipitated DNA was performed using SYBR green and specific ChIP primers for TNF-α and IL-6 promoters. The primers used for ChIP were described in the previous study (27). Results are presented as % of input. The IgG control did not yield a signal in the PCR analysis.

### Mice and BAFF Treatment

All the animal experiments were carried out according to the Animal Care and Use Committee of China Medical University. Male C5B7L/6J mice were housed in a humidity-controlled (50–55%) and temperature-controlled (22–24°C) facility on a 12 h light/dark cycle (lights on at 7:30 am) with free access to food (standard laboratory rat chow) and water.

Male mice at 9 weeks of age were randomly divided into three groups: (1) sham group (ASCF+ASCF, n = 12); (2) unprimed group (ASCF+BAFF, n = 12); (3) primed group (BAFF+BAFF, n = 12). Mice were treated with either BAFF (BAFF priming, 0.5 μg/ml dilution in ACSF) or artificial cerebrospinal fluid (ASCF) by intracerebroventricular injection (ICV) and 7 days later with BAFF restimulation (1 μg/ml). The behavioral test was performed 24 h after the last ICV injection. After completing all the behavioral tests, the mice were sacrificed, and the brain was removed and stored at −80°C until use.

For ICV, mice were anesthetized and placed in a stereotaxic frame (DW-2000, Taimeng, Chengdu, China) under sterile conditions, and after an incision in the skin, the cranium was exposed. And small holes were drilled over the parietal bone for injection. Guide cannula was implanted in lateral ventricle using stereotactic co-ordinates (AP = −0.3 mm, ML = 1.0 mm, and DV = −2.5 mm) (28). The guide cannula was encased with a dummy cannula to help maintain sterility. Five days after surgery, ICV injection of BAFF or artificial cerebrospinal fluid (ACSF; glucose, 5 mM; CaCl_2_, 1 mM; NaCl, 125 mM; MgCl_2_, 1 mM; NaHCO_3_, 27 mM; KCl, 0.5 mM; Na_2_SO_4_, 0.5 mM; NaH_2_PO_4_, 0.5 mM; and Na_2_HPO_4_, 1.2 mM). A 24-gauge needle connected with polyethylene tubing to a 10-μl Hamilton microsyringe (P/N:80300/00, Hamilton, USA) was used for ICV injection. An injection volume of 2 μl was delivered using a syringe pump (Harvard Apparatus, Holliston, MA, USA) at a rate of 0.2 μl/min. The injection needle remained in place for at least 10 min after the infusion before being pulled out to prevent backflow of the injection.

### Immunohistochemistry

After completing the behavioral tests, the mice were anesthetized and perfused with 80 ml of ice-cold 0.9% saline followed by 60 ml of 4% paraformaldehyde. The brain was taken out and post-fixed with the same fixative overnight at 4°C, and then the brain was treated with 30% sucrose at 4°C for 2 days. Brain tissue was sectioned and pasted into microscope slides. Brain sections were blocked for 30 min at room temperature incubation in PBS containing 10% donkey serum. The sections were incubated overnight with Iba1 antibody at 4°C, then were washed in PBS and incubated with fluorescent secondary antibody, fluorescein isothiocyanate (FITC)-conjugated Donkey anti-Rabbit IgG (H+L) (1:1000, Proteintech) for 1 h at room temperature. The images were captured using the Nexcope NIB410 microscope system and analyzed with Image J software. Representative bright-field images were obtained using a 20× objective lens.

### Brain Tissue Cytokine and Chemokines Measurement

Mice were anesthetized, and the brain was rapidly removed. The brains were dissected in PBS containing protease inhibitors on ice and homogenized. To remove the debris, the supernatants were harvested by centrifugation. Total protein content was determined with BCA assay. Cytokine or chemokine concentration in the supernatants were determined by ELISA. The concentration of cytokines or chemokines was normalized to each sample’s protein concentrations and described as ng/g of total protein.

### Statistical Analysis

All statistical analyses were conducted using the SPSS 23 software (IBM Inc, New York, USA). All data were expressed as the mean ± SEM. Continuous values among groups were analyzed using the non-parametric Kruskal-Wallis test with Turkey *post hoc* test. A level of 5% was used to define statistical significance (p < 0.05).

## Results

### BAFF Induced a Trained Immunity-Like Phenotype of BV2 Cells

The concept of trained immunity describes an activated monocyte phenotype that enables an enhanced inflammatory cytokine production in response to a secondary challenge such as TLR agonist or bacterial infections (29, 30). In our experiments, we used BV2 cells as a cell model of microglia. The *in vitro* experiments are graphically described in **Figure 1A**. BV2 cells were treated with vehicle (cellular culture medium) or primed with different doses of mouse recombinant BAFF (2.5, 5, 10ng/ml, described as “BAFF priming” in the figures) for 24 h and rested for 5 days. On day 6, BV2 cells were restimulated with LPS (described as “LPS restimulation” in the figures) or a higher dose of BAFF (40 ng/ml, described as “BAFF restimulation” in the figures) for 24 h. Consist with circulatory monocytes, BAFF priming induced potent proinflammatory cytokines (TNF-α, IL-6, and IL-1β) secretion upon LPS stimulation on day 7 (**Figures 1B–D** **;** p < 0.001). This phenomenon is likely to accord with myeloid cells’ trained immunity phenotype induced by β-glucan, ox-LDL, or an ultra-low dose of LPS (2, 29, 31), indicating that BAFF itself was able to establish a memory-like inflammatory response of microglia. Of note, restimulation with BAFF instead of LPS on day 6 yielded less potent but corresponding results of LPS stimulation (**Figures 1E–G**; p < 0.001), indicating that BAFF priming resulted in a memory response of BV2 cells to both LPS and BAFF itself. In terms of the elevated BAFF in the CSF of chronic neuroimmunological diseases, low-dose BAFF priming may elicit a “BAFF” signature in microglial cells in CNS, enabling microglial cells to be more sensitive to BAFF restimulation or infection.

Due to the influence of BAFF on cell proliferation (32), we examined the cell viability and proliferation by MTT and Trypan blue assay, respectively, on day 7. As **Supplementary Figure 1** showed, BAFF training and LPS training elicited no noticeable effect on viability and proliferation of BV2 cells (p >0.05). Besides, without restimulation with BAFF or LPS, TNF-α, IL-6, and IL-1β levels were not up-regulated following BAFF priming (**Supplementary Figure 2**, p >0.05), confirming the training effect of BAFF and LPS.

Taken together, these results all above confirmed that BAFF itself induced a training effect on microglia in a dose-dependent manner.

### Glycolysis Was Essential for BAFF-Inducing Microglial Training

**Figure 2:**
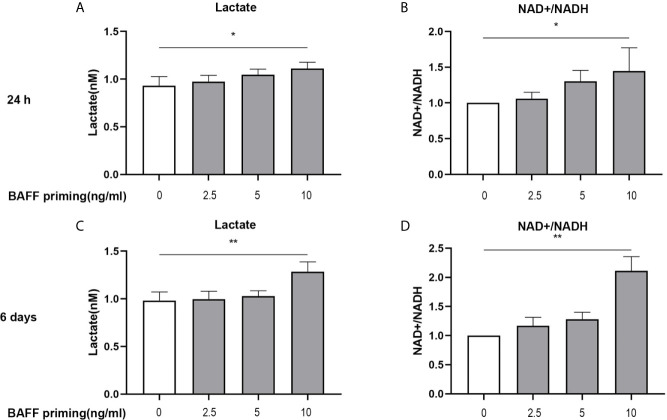
BAFF priming was associated with the induction of glycolysis. BV2 cells were primed with BAFF or vehicle for 24 h and then rested for 5 days. The production of lactate in the supernatants (n = 12) and NAD+/NADH ratio (n = 12) in the whole-cell lysates were assayed 24 h after BAFF priming **(A, B)** and on day 6 before restimulation **(C, D)** respectively. Data are means ± SED; Kruskal-Wallis test with Turkey *post hoc* test; *p < 0.05 and **p < 0.01 compared with CTL.

In addition to a robust pro-inflammatory activation, another characterization of trained immunity is enhanced aerobic glycolysis of the trained cells. On day 1 (24 h after BAFF priming) and day 6 (before restimulation), the glycolytic level of BV2 cells was measured. As shown in **Figure 2**, upon 10 ng/ml BAFF priming, BV2 cells showed more lactate in cellular supernatants and an increased ratio of NAD+/NADH in cell lysates on 24 h (**Figures 2A, B** **;** p< 0.05). On day 6, BAFF priming enhanced lactate production and NAD+/NADH ratio even after removing BAFF in the cellular supernatants, indicating a sustained enhanced glycolytic capacity in BAFF-primed BV2 (**Figures 2C, D**; p< 0.01).

**Figure 3:**
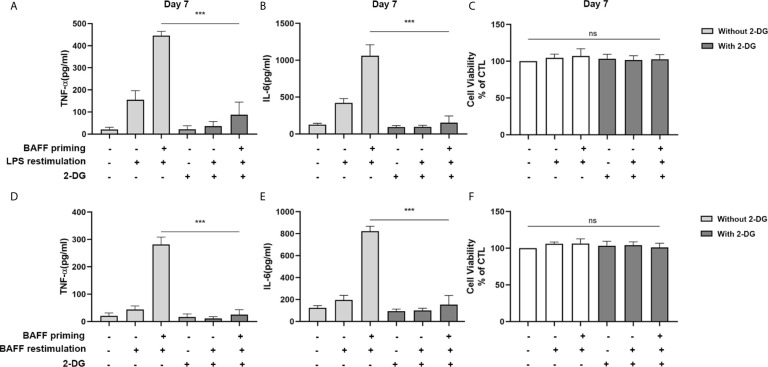
Inhibition of glycolysis by 2-DG blocked proinflammatory cytokines secretion of BAFF-training BV2 cells. BV2 cells were pretreated with 1mM 2-DG for 1 h, then primed with vehicle or BAFF for 24 h. After a rested of 5 days, cells were restimulated with LPS or BAFF for 24 h. The amounts of TNF-α **(A)** and IL-6 **(B)** were measured in the culture supernatants and the cytotoxicity of 2-DG was measured by MTT **(C)** on day 7 upon LPS restimulation (n = 12). The secretion of TNF-α **(D)** and IL-6 **(E)** and the cytotoxicity of 2-DG **(F)** were measured on day 7 upon BAFF restimulation (n = 12). The Data are mean ± SEM; Kruskal-Wallis test with Turkey *post hoc* test; ***p < 0.001. ns: no significance between groups.

Accordingly, the role of glycolysis in initiating and maintaining microglial training was further confirmed by the incubation of cells with 2-deoxy-d-glucose (2-DG), a glucose analog that inhibited glycolysis (32). As depicted in **Figures 3A, B**, 1 mM 2-DG pre-treatment inhibited the expression of proinflammatory cytokines TNF-α and IL-6 induced by BAFF priming (10 ng/ml) and LPS restimulation (p < 0.001). Also, 2-DG displayed a similar inhibitory effect on BAFF training cells (**Figures 3D, E**; p< 0.001). 2-DG used here did not affect the cell viability of BV2 cells (**Figures 3C, F**). These data demonstrated that metabolic reprogramming toward aerobic glycolysis is essential for regulating BAFF-induced microglial training.

### BAFF Priming Induces an Epigenetic Trained Immunity Phenotype in BV2 Cells

**Figure 4:**
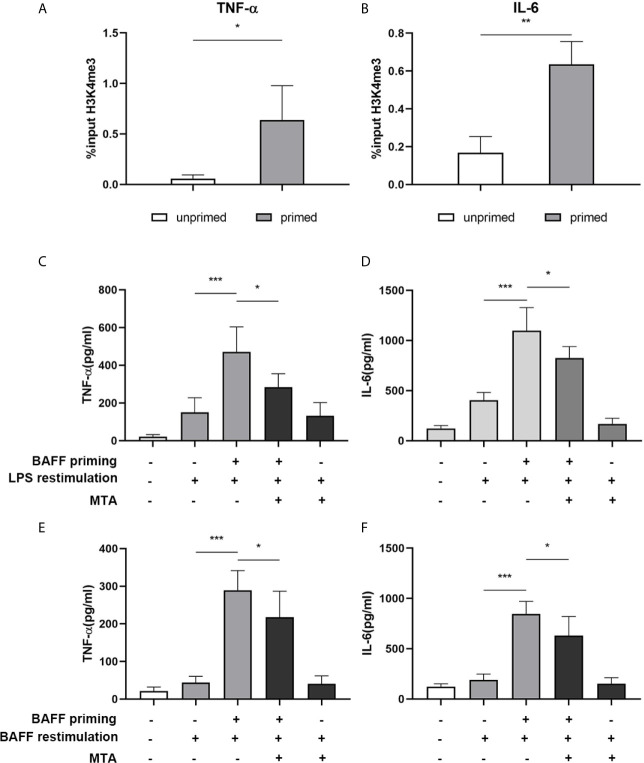
BAFF priming induces an epigenetic trained immunity phenotype in BV2 cells. **(A, B)** BV2 cells were treated with 10ng/ml BAFF or vehicle for 24 h and kept for 5 days. ChIP assay was performed using an antibody against histone 3 lysine 4 trimethylation (H3K4me3) or control IgG. The enrichment of H3K4me3 at the promoters of TNF-α **(A)** and IL-6 **(B)** was quantified by quantitative real-time PCR. The results are expressed as % input and data are means ± SED (n = 6); t-test; *p < 0.05. **(C, D)** BV2 cells were treated as indicated with 10 ng/ml BAFF, 20 mM MTA (histone methyltransferase inhibitor) or vehicle for 24 h, rested for 5 days, on day 6 treated with LPS restimulation for 24 h. TNF-α **(C)** and IL-6 **(D)** were measured in the supernatants by ELISA on day 7(n = 12). On day 6, BV2 cells were restimulated with BAFF instead of LPS. On day 7, TNF-α **(E)** and IL-6 **(F)** were measured in the supernatants (n = 12). Data are means ± SED (n = 12); Kruskal-Wallis test with Turkey *post hoc* test; *p < 0.05, **p < 0.01, and ***p < 0.001.

Long-term proinflammatory memory during trained immunity is based on persistent epigenetic modifications on the promoters of inflammatory genes, including trimethylation at lysine 4 of histone H3 (H3K4me3) (27). Therefore, we investigated whether BAFF priming induced similar epigenetic changes in mouse microglial cells. As demonstrated in **Figures 4A, B**, BAFF priming resulted in a significant increase of the activating histone modifications H3K4me3 on the TNF-α (p< 0.05) and IL-6 (p< 0.01) promoter. We further investigated the link between proinflammatory effects and epigenetic changes using the epigenetic inhibitor MTA (methylthioadenosine, a methyltransferase inhibitor). As demonstrated in **Figures 4C–F**, MTA significantly inhibited proinflammatory cytokine production induced by BAFF training (p< 0.05).

### TACI and Akt/mTOR/HIF-1α Were Involved in BAFF Priming of BV2 Cells

**Figure 5:**
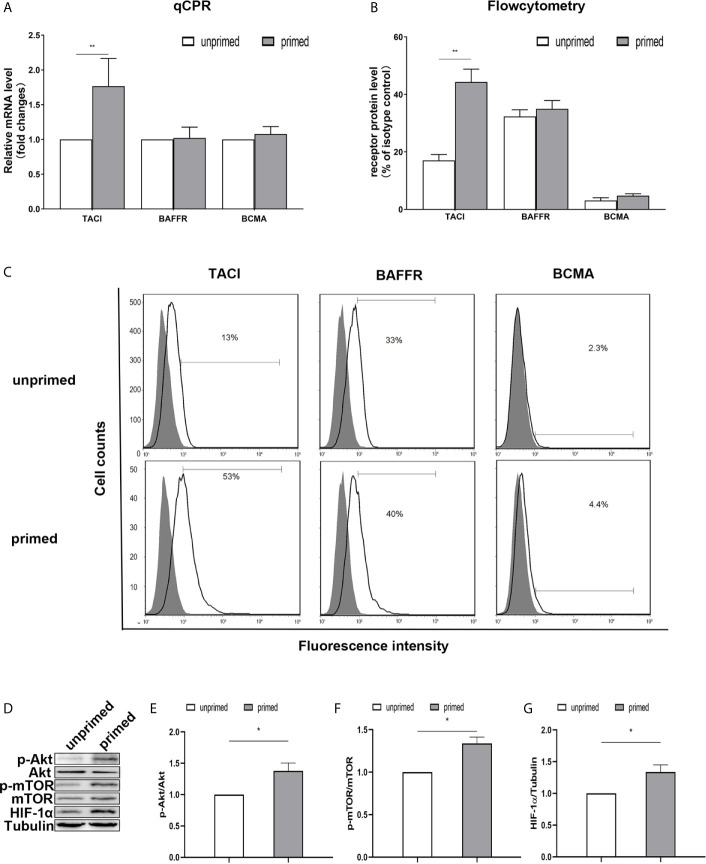
TACI and Akt/mTOR/HIF-1α signaling were involved in BAFF priming of BV2 cells. BV2 cells were primed with 10ng/ml BAFF or vehicle for 24 h. **(A)** RNA was collected and the relative mRNA expression of TACI, BAFF-R, and BCMA, relative to GAPDH, are assessed in BV2 cells by quantitative real-time PCR. **(B, C)** The expression of BAFF receptors on the surface of microglia was detected by flow cytometry. BV2 cells are stained with primary antibodies for TACI, BAFF-R, and BCMA (solid lines without filling) or an isotype control Ab (solid lines filled with gray). **(B)** The graph represents the relative fluorescence intensity of the primary antibody subtracting its isotype control antibody. **(D)** 6 days after BV2 cells were primed by BAFF or vehicle, whole-cell extracts were collected and levels of Akt, p-Akt, mTOR, p-mTOR, and HIF-1α were detected by western blot. Densitometric quantification of p-Akt/Akt **(E)**, p-mTOR/mTOR **(F)**, and HIF-1α/tubulin **(G)** in **(D)**. Data are mean ± SEM (n = 9); Kruskal-Wallis test with Turkey *post hoc* test; *p < 0.05, **p < 0.01 compared with CTL.

Previous studies have found the expression of BAFF receptors on the surface of mice microglia (19). Kim et al. (19) demonstrated that BV-2 cells constitutively expressed BAFF-R on the cell surface and BAFF-R medicated microglial activation induced by brief exposure of BAFF *in vitro*. Here, we used a real-time PCR assay to measure the mRNA expression of TACI, BAFFR, and BCMA in the BV2 microglia. On day 1, BAFF priming only increased TACI expression (**Figure 5A**; p< 0.01). However, we observed no difference in the expression of TACI between the primed and unprimed BV2 cells on day 6 (**Supplementary Figure 3**). Next, we used flow cytometry to examine the BAFF receptors’ expression on BV2 cell surface on day 1. We found a more vital expression of TACI and BAFFR and relatively weaker expressions of BCMA on the BV2 cell surface (**Figure 5C**), which corresponded with the results of the previous study (19). The expression of TACI was up-regulated upon BAFF stimulation (**Figures 5B, C**, p< 0.01). To clarify TACI’s roles in BAFF-induced training responses, we used a siRNA approach to knock down TACI. As shown in **Supplementary Figure 4**, TACI expression was inhibited by TACI siRNA (TACI siRNA) but not by control siRNA (NC-siRNA). Compared with NC-siRNA, TACI siRNA significantly downregulated the secretion of TNF-α and IL-6 upon LPS restimulation or BAFF restimulation (**Supplementary Figures 5D, E**). All the data indicated that BAFF induced a proinflammatory trained-immunity phenotype through TACI.

TACI activation recruits mTOR, and ensuing activation of mTOR drives immune responses by linking TACI signaling with immunometabolic signaling (33). It is well established that Akt/mTOR/HIF-1α pathway mediates the development of trained immunity in circulatory monocytes (29). Next, we examined the expression of the Akt/mTOR/HIF-1α pathway in BV2 cells induced by BAFF priming. Compared with unprimed cells, BAFF-priming cells exhibited increased expression of p-Akt/Akt ratio, p-mTOR/mTOR ratio, and HIF-1α assayed by Western blot (**Figures 5D–G**; p< 0.05), which is in line with the increase of glycolytic capacity. The data above suggested the involvement of the Akt/mTOR/HIF-1α pathway in the BAFF training of BV2 cells.

### Blockade of Akt/mTOR/HIF-1α Inhibited the BAFF-Training of BV2 Cells

**Figure 6:**
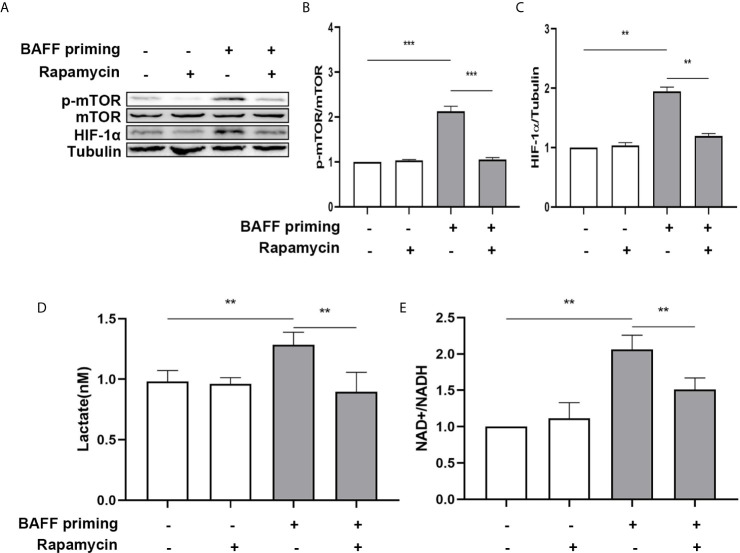
BAFF-induced metabolic reprogramming was blocked by rapamycin in BV2 cells. **(A)** On day 0, BV2 cells were pretreated with 100 nM rapamycin for 1 h and then treated with vehicle or BAFF (10 ng/ml) for 24 h, rested for 5 days. BV2 cells were lysed on day 6. Whole-cell extracts were subjected to western blot for the expression level of mTOR, p-mTOR, and HIF-1α. Densitometric quantification of p-mTOR/mTOR **(B)** and HIF-1α/tubulin **(C)** in **(A)** (n = 9). **(D)** Lactate was measured in the culture supernatants on day 6 (n = 12). **(E)** The ratio of NAD+/NADH was detected in whole-cell lysates on day 6 (n = 9). Data are mean ± SEM; Kruskal-Wallis test with Turkey *post hoc* test; **p < 0.01, and ***p < 0.001.

mTOR is required for metabolic reprogramming, which is necessary for monocytes’ trained immunity phenotype (2, 29). Therefore, we analyzed the contribution of mTOR in BAFF-induced inflammatory priming. Rapamycin (Sirolimus, AY 22989) is a potent and specific mTOR inhibitor that binds to FKBP12 and acts explicitly as an allosteric inhibitor of mTORC1 (34). As demonstrated in **Figures 6A–C**, pharmacologic inhibition of mTOR with rapamycin blocked the phosphorylation of mTOR (**Figure 6B**; p< 0.001) and expression of HIF-1α (**Figure 6C**, p< 0.01) in BAFF-priming BV2 cells. Moreover, rapamycin effectively suppressed the enhanced glycolysis of BV2 cells caused by BAFF indicated with reduced lactate production (**Figure 6D**; p< 0.01) and NAD+/NADH (**Figure 6E**; p< 0.01), suggesting that mTOR-dependent HIF-1α accumulation is a prerequisite for the profound metabolic reprogramming during trained innate immunity (29, 35).

**Figure 7:**
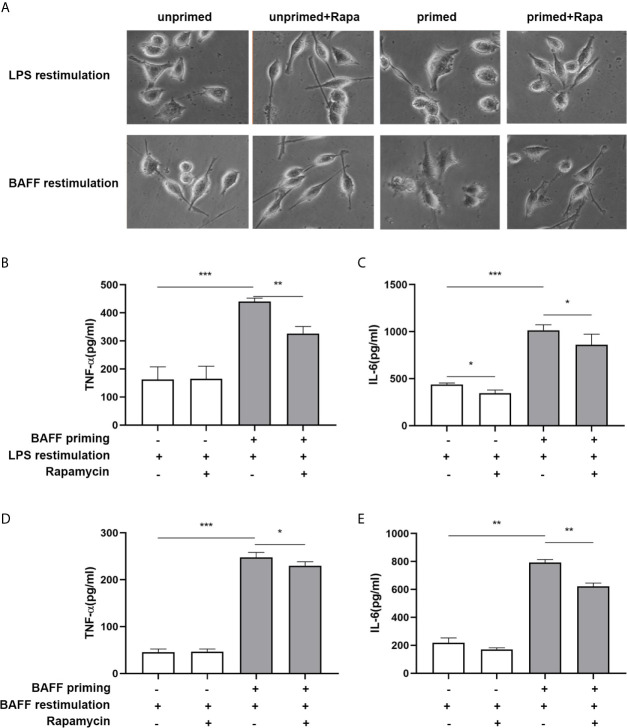
Rapamycin inhibited BAFF-induced trained immunity phenotype of BV2 cells. BV2 cells were pretreated with 100 nM rapamycin for 1 h, then primed with vehicle or BAFF for 24 h. After a rested of 5 days, cells were restimulated with BAFF or LPS for 24 h. **(A)** Representative micrographs of BV2 cells on day 7(n = 9). The amounts of TNF-α **(B)** and IL-6** (C)** were measured in the culture supernatants on day 7 upon LPS restimulation (n = 12). The amounts of TNF-α **(D)** and IL-6 **(E)** were measured in the culture supernatants on day 7 upon BAFF restimulation (n = 12). Data are mean ± SEM; Kruskal-Wallis test with Turkey *post hoc* test; *p < 0.05, **p < 0.01, and ***p < 0.001.

In addition to metabolic changes, we also estimated morphological changes and proinflammatory response induced by BAFF training. The morphologic changes and proinflammatory cytokine production of BV2 cells were assayed on day 7. As depicted in **Figure 7A**, BAFF training induced an evident morphological activation of BV2 cells upon LPS stimulation indicated by amoeboid shape or larger cell body with process contraction. Rapamycin restored the activated BV2 cells to a more petite cell body. Results of the ELISA assays demonstrated that rapamycin impaired secretion of TNF-α (**Figure 7B**; p< 0.01) and IL-6 induced by BAFF training (**Figure 7C** **;** p< 0.05). We also assayed whether rapamycin could affect the proinflammatory memory of BV2 cells upon BAFF restimulation. As demonstrated in **Figures 7A, D, E**, rapamycin intervention elicited similar effects on BAFF-inducing training all by itself.

**Figure 8:**
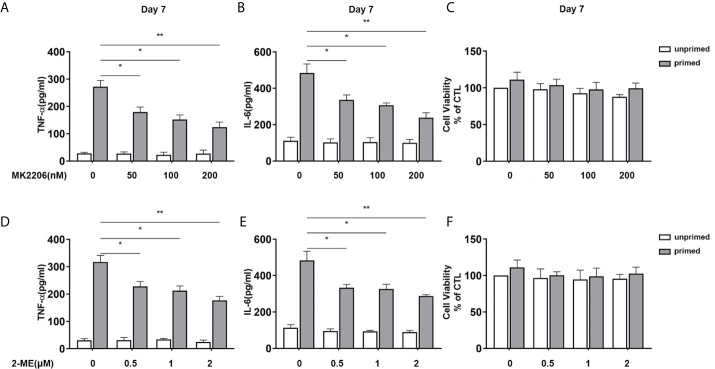
Inhibition of Akt or HIF-1α suppressed BAFF training of BV2 cells in a dose-dependent manner. **(A–C)** BV2 cells were pretreated with different doses of MK2206, a specific Akt inhibitor (50, 100, 200 nM) for 3 h, and then primed with BAFF for 24 h. After a rested of 5 days, cells were restimulated with BAFF for 24 h. The amounts of TNF-α **(A)** and IL-6 **(B)** were measured in the culture supernatants and the cytotoxicity of MK2206 was measured by MTT **(C)** on day 7(n = 12). **(D-F)** BV2 cells were pretreated with different doses of 2-Methoxyestradiol (2-ME), a specific HIF-1α inhibitor (0.5, 1, 2 μM) for 3 h, and then primed with BAFF for 24 h. The secretion of TNF-α **(D)** and IL-6 **(E)** and the cytotoxicity of 2-ME **(F)** were measured on day 7 upon BAFF restimulation (n = 12). The data are mean ± SEM; Kruskal-Wallis test with Turkey *post hoc* test; *p < 0.05, **p < 0.01.

Apart from mTOR, we also inhibited Akt and HIF-1α with their specific inhibitor and evaluated their influence on the proinflammatory trained immunity phenotype induced by BAFF. BV2 cells were pre-incubated with Akt inhibitor MK2206 (Selleck Chemicals, USA, S1078) or HIF1-α inhibitor 2-Methoxyestradiol (2-ME) (MCE, USA, HY-12033) for 3 h and then primed by 10 ng/ml BAFF. On day 7, MK2206 abolished the IL-6 and TNF-α secretion of BV2 upon both BAFF restimulation (**Figures 8A, B** **)** or LPS restimulation (**Supplementary Figures 6A, B**). As expected, HIF1-α inhibitor 2-ME also suppressed the secretion of these proinflammatory cytokines in BAFF-training BV2 cells (**Figures 8D, E** and **Supplementary Figures 6D, E**). MTT was performed to exclude the potential influence of cell viability on cytokine secretion. As expected, MK2206 and ascorbate used here did not affect the cell viability of BV2 cells (**Figures 8C, F** and **Supplementary Figures 6C, F**). Moreover, the anti-proinflammatory effects of MK2206 or 2-ME displayed in a dose-dependent manner, asserting the essential roles of Akt/mTOR/HIF-1α pathway in the BAFF-inducing trained immunity-like phenotype.

### Inhibition of mTOR by Rapamycin in Primary Microglia Suppressed Trained Immunity Phenotype Induced by BAFF

Next, we investigated whether BAFF elicited a proinflammatory trained immunity phenotype of mouse primary microglia and whether mTOR inhibition by rapamycin exerted similar effects on primary microglial cells.

First of all, we performed real-time PCR to examine BAFF receptors’ expression in mouse primary microglial cells. Of the three receptors, only TACI was up-regulated by BAFF priming (10 ng/ml) on day 1 (**Supplementary Figure 7A**, p< 0.05) in mouse primary microglial cells. However, there was no apparent difference between the mRNA expression between the primed and unprimed one on day 6 (**Supplementary Figure 7B**, p>0.05). Similar to BV2 cells, TACI was the downstream receptor for BAFF training in mouse primary microglial cells.

**Figure 9:**
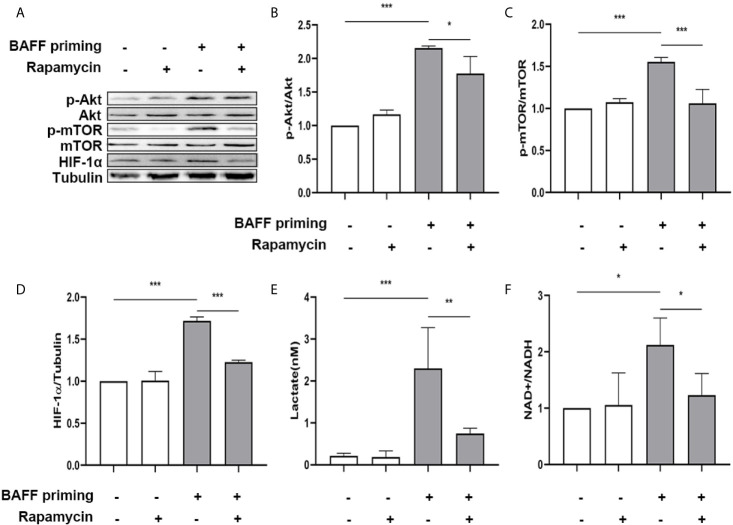
Rapamycin suppressed HIF-1α and metabolic reprogramming in primary microglial cells induced by BAFF priming. Primary microglia were pretreated with 100 nM rapamycin for 1 h, then treated with BAFF (10 ng/ml) or vehicle for 24 h, and rested for 5 days. **(A)** Whole-cell extracts were collected on day 6 and levels of Akt, p-Akt, mTOR, p-mTOR, and HIF-1α were detected by Western blot. Densitometric quantification of p-Akt/Akt **(B)**, p-mTOR/mTOR **(C)**, and HIF-1α/tubulin **(D)** in **(A)**. **(E)** The production of lactate in the supernatants was measured on day 6; **(F)** The ratio of NAD+/NADH in the whole-cell lysates was measured on day 6. Data are mean ± SEM (n = 9); Kruskal-Wallis test with Turkey *post hoc* test; *p < 0.05, **p < 0.01, and ***p < 0.001.

Next, primary microglial cells were pretreated with 100 nM rapamycin for 1 h before exposure to BAFF priming and then rested for 5 days. We investigated whether rapamycin was able to suppress HIF-1α and metabolic reprogramming induced by BAFF priming. In accordance with BV2 cells, BAFF priming caused the activation of Akt/mTOR/HIF-1α signaling detected by western blot on day 6. Rapamycin was sufficient to inhibit the activation of mTOR (**Figure 9C**; p< 0.001) and its downstream HIF-1α (**Figure 9D**; p< 0.001), as shown in **Figures 9A–D**. In line with Akt/mTOR/HIF-1α signaling pathway, rapamycin suppressed the overproduction of lactate (**Figure 9E**; p< 0.01) and elevated NAD+/NADH (**Figure 9F**; p< 0.05) induced by BAFF.

**Figure 10:**
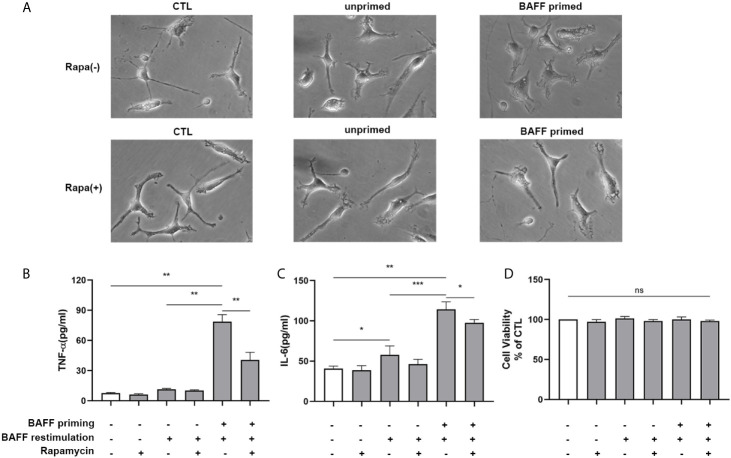
Rapamycin suppressed proinflammatory memory-like state of primary microglial cells induced by BAFF training. Primary microglia were pretreated with 100 nM rapamycin for 1 h and then treated with BAFF (10 ng/ml) or vehicle for 24 h. On day 6, microglial cells were restimulated with BAFF (40 ng/ml) for 24 h. **(A)** The representative micrographs of primary microglial cells on day 7. The amounts of TNF-α **(B)** and IL-6 **(C)** were measured in the culture supernatants on day 7. **(D)** Cell viability of primary microglial cells on day 7 was tested by MTT assay. Data are mean ± SEM (n = 9); Kruskal-Wallis test with Turkey *post hoc* test; *p < 0.05, **p< 0.01, and ***p< 0.001, ns: no significance between groups.

Next, we examined whether rapamycin alleviated a proinflammatory memory-like state induced by BAFF training. Since neuroinflammation involved in neuroimmunological diseases is a mainly sterile inflammatory response rather than infections, primary mouse microglial cells were restimulated with 40 ng/ml BAFF on day 6. 24 h after the restimulation of BAFF, the morphological changes and proinflammatory cytokine production were estimated. As manifested in **Figure 10**, the unprimed primary microglial cells displayed shortened cellular branches. In contrast, the BAFF-priming microglial cells showed an obvious enlarged cell body with thickening and retraction of cellular branches, which was defined as proinflammatory morphological activation (25, 36). Microglial cells pretreated with rapamycin transformed into a morphology similar to the unprimed one. Besides, primary microglial cells primed by BAFF did secrete a higher amount of TNF-α (**Figure 10B**, p< 0.01) and IL-6 (**Figure 10C**; p< 0.001) compared with unprimed microglial cells. In addition to proinflammatory cytokines, we also examined the expression of proinflammatory chemokines CCL2. As shown in **Supplemental Figure 8**, BAFF training did increased the secretion of CCL2 compared with the unprimed one (p<0.001). Rapamycin suppressed the secretion of these cytokines (**Figure 10B**; p< 0.01 and **Figure 10C**; p<0.05) and chemokines (**Supplementary Figure 8**, p<0.01). BAFF and rapamycin had no obvious influence on primary microglial cells’ cell viability (**Figure 10D**; p >0.05). Consistent with the results from BV-2 cells, these experiments support that BAFF led to a trained immunity-like phenotype of primary microglial cells indicated with morphological activation, robust pro-inflammatory cytokines secretion, and enhanced glycolysis. Inhibition of mTOR by rapamycin was sufficient to block the proinflammatory activation and metabolic reprogramming of primary microglial cells induced by BAFF.

### BAFF Induced Microglial Training *In Vivo*

**Figure 11:**
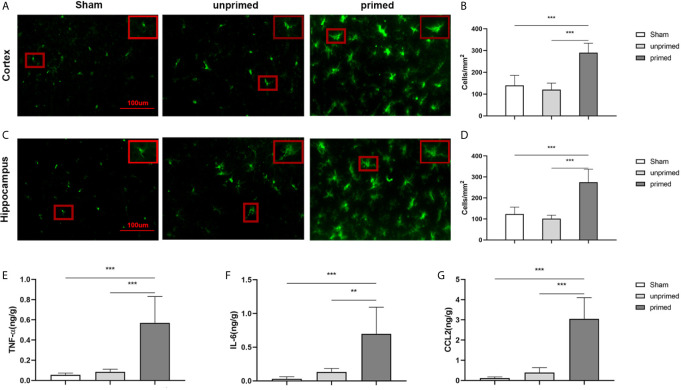
BAFF induced microglial training *in vivo*. Immunofluorescence staining of Iba1 in the cortex **(A)** and hippocampus **(C)**. Comparisons of the numbers of Iba1-positive microglia in the cortex **(B)** and hippocampus **(D)** (n = 6). A representative Iba-1+ cell from the cortex is shown magnified in the red box in the image’s right upper corner. The expression of TNF-α **(E)**, IL-6 **(F)**, CCL2 **(G)** were measured in the supernatants by ELISA on day 7(n = 6). Data are mean ± SEM; Kruskal-Wallis test with Turkey *post hoc* test; **p < 0.01, and ***p < 0.001.

The mice were randomly divided into three groups: sham group (ASCF+ACSF), unprimed group (ASCF+BAFF), and primed group (BAFF+BAFF). To evaluate whether locally elevated levels of BAFF within the CNS could induce microglial training, Iba-1 staining was performed on the brain sections to evaluate the number and morphology of microglia. For BAFF priming, mice were treated with either BAFF (BAFF priming) or artificial cerebrospinal fluid (ASCF) by intracerebroventricular injection and 7 days later with BAFF restimulation. 24 h after BAFF restimulation, the number of Iba-1 microglia in the cortex and hippocampus were significantly increased in the primed group compared with those unprimed group and the sham group (**Figures 11A–D**). Specifically, the dominant morphology of microglia observed in the primed group was larger cell bodies and thicker processes (**Figures 11A, C**, magnified red box).

We further used ELISA to measure the expression of proinflammatory cytokines and chemokine in the brain tissue. As displayed in **Figure 11**, levels of TNF-α (**Figure 11E**), IL-6 (**Figure 11F**), and CCL2 (**Figure 11G**) were increased in the primed group compared with those in the unprimed group and sham group. All the results confirmed that BAFF priming induced microglial immune training in the brain.

## Discussion

A growing body of evidence suggests that “trained” microglia contributes to the pathogenesis of chronic neuroinflammation (5, 31). In the present study, we provided proof-of-principle for BAFF-inducing trained immunity-like phenotype in microglia *in vitro* and *in vivo* for the first time. In our experiments, the immune memory of microglia developed upon the BAFF priming indicated by long-term enhanced glycolysis. The effects of immune memory to BAFF became most apparent following LPS stimulation, corroborating the concept of “trained immunity” (29). Moreover, BAFF priming activated Akt/mTOR/HIF-1α signaling pathway in microglia. Inhibition of mTOR blocked the metabolic rewiring as well as the proinflammatory memory-like response of microglia. It is worth noting that the BAFF-priming BV2 cells and primary mouse microglial cells displayed a similar activation pattern upon BAFF restimulation, indicating that low-dose BAFF priming enables microglial cells to be highly responsive to either BAFF restimulation or infection. Intriguingly, repeated BAFF exposure in the CNS did exert more obvious microglial activation *in vivo* and up-regulation of proinflammatory cytokines and chemokine in the brain, which verified the training effect of microglial cells induced by BAFF.

The “trained immunity” is an emerging phenomenon described mainly in peripheral innate immune cells, including monocytes and tissue-resident macrophages. The priming or trained cells develop a “memory” to inflammatory and infectious stimuli through metabolic and epigenetic reprogramming (37). As the only parenchymal macrophages of the CNS, microglia is highly plastic and is known to be pre-activated or “primed” by some inflammatory processes. Recent investigations brought trained immunity of microglia into the spotlight. Peripherally applied LPS or cytokines (IL-1β, TNF-α) evokes microglial activation and neuroinflammation in the brain, which exacerbates cerebral β-amyloidosis in a mouse of Alzheimer’s pathology (5). Besides, an ultra-low dose of LPS is able to prime microglia with enhanced phagocytosis upon LPS restimulation (31). Likewise, we demonstrated that both BV2 cells and primary mouse microglial cells primed by BAFF were equipped with an enhanced inflammatory response to LPS and BAFF itself indicated by amoebic shape and robust pro-inflammatory cytokines secretion. On the other hand, trained immunity is characterized by metabolic reprogramming from oxidative phosphorylation (OXPHOS) to glycolysis (29). In our study, the BAFF-training effect is confirmed by long-lasting enhanced aerobic glycolysis in microglial cells indicated by over-production of lactate and increased ratio of NAD+ to NADH. Glycolysis enables innate immune cells to fulfill the energy-intensive consumption which is needed in cellular immune responses such as migration, cytokine secretion, and phagocytosis. Upon activation, microglia acquires a transient shift from OXPHOS to aerobic glycolysis, which is known as the Warburg effect (38). Distinct from the temporary metabolic shift, trained immunity signed a long-term metabolic reprogramming to primed cells that prepare the primed cells to respond to future challenges robustly and rapidly. Our data reveals the relationship of metabolic shift and trained immunity phenotype of microglial cells in line with previous research (5).

To explore the potential mechanism of BAFF training on microglial cells, we estimated the possible signaling pathway activated following BAFF training. In addition to the classic signaling pathway nuclear factor kappa-light-chain-enhancer of activated B cells (NF-κB), BAFF has been confirmed to activate Akt/mTOR signaling pathway for promoting B cell proliferation (32). This process is possibly mediated by TACI, which is able to recruit mTOR through Myd88 (33). Besides, Akt/mTOR/HIF-1α signaling is a well-established signaling pathway involved in circulatory monocytes‘ trained immunity (29). With this regard, we examined the expression of BAFF receptors and whether BAFF priming could activate Akt/mTOR/HIF-1α signaling. In our present study, we demonstrated BAFF priming induced a transient expression of TACI on BV2 cells, and Akt/mTOR/HIF-1α is activated on day 6 after BAFF priming, which is in line with the observations of trained immunity in monocytes (2, 29). Knock-down of TACI with siRNA abolished the training effect of BAFF on microglia, indicating the critical role of TACI in the training immunity induced by BAFF. Pharmacological blockage of mTOR by specific mTOR1 inhibitor rapamycin was sufficient to inhibit the downstream HIF-1α accumulation, which is a transcription factor that regulates various genes associated with glycolysis as well as proinflammatory cytokines such as IL-1β, IL-6 (39–41). Accordingly, rapamycin reduced the glycolysis as well as the proinflammatory activation of microglia induced by BAFF priming. Additionally, inhibition of Akt and HIF-1α with its corresponding inhibitor was sufficient to suppress the BAFF training. Most importantly, rapamycin effectively alleviated the BAFF-inducing microglia *in vivo*. Taken together, Akt/mTOR/HIF-1α cell signaling mediates the development of the trained immunity phenotype of microglia.

It is well acknowledged that long-term proinflammatory memory during trained immunity is based on persistent epigenetic modifications on the promoters of inflammatory genes, such as mono-methylation at lysine 4 of histone 3 (H3K4me1), trimethylation at lysine 4 of histone H3 (H3K4me3), and acetylation at lysine 27 of histone 3 (H3K27ac) (1, 37). Accumulation of glycolysis or tricarboxylic acid (TCA) cycle metabolites will, in turn, shape the immune response by epigenetic modifications. Trained monocytes accumulate fumarate upon β-glucan, which decreases the activity of KMD5 histone demethylases and consequently promotes the expression of genes involved in the pro-inflammatory response (30). Another metabolite, succinate, is able to facilitate the stabilization of HIF-1α (40, 42).

Our study focused on H3K4me3 and, for the first time, confirmed the role of a sustained H3K4me3 modification on the promoters of proinflammatory cytokines during the BAFF training. Moreover, a methyltransferase inhibitor partially modifies the induction of proinflammatory cytokine production in the trained microglial cells, which provides a new target for neuroimmunological diseases. However, whether and how BAFF training influences epigenetic processes and the cross-link of epigenetic modifications and metabolic reprogramming in trained immunity are still under further investigation.

The training responses of microglia primed with BAFF may raise the question of the pathological effect of BAFF in CNS. Here, we used the ICV administration of BAFF in mice to mimic the locally elevated BAFF level in CNS. This model also provides a pathological environment to analyze brain-resident cells’ change without the involvement of cells from peripheral circulation such as monocytes or macrophages. Our study confirmed that microglia were activated by double BAFF injection indicated by microgliosis and morphological changes in the cortex and hippocampus. Besides, BAFF training-induced proinflammatory cytokines and chemokine expression including TNF-α, IL-6, and CCL2 in brain tissue, which in line with the findings of primary microglial cells. With this regard, our results raise the possibility that BAFF training may play an essential role in the pathogenesis of neuroinflammatory diseases. However, how the BAFF training causes the observed pathophysiological aberration and the cross-talk between microglia and neuron are still need further research for their elucidation.

Our current findings firstly highlight the importance of BAFF in chronic neuroimmunological diseases. In our study, a relatively low dose of BAFF was able to elicit a priming effect on microglial cells *in vitro*, which prepared the brain-resident microglial cells to respond to another pulse of BAFF stimulation in an enhanced manner. In terms of the vital role of microglia in neuroinflammation, this “BAFF signature” would lead to persistent inflammation, which contributes to chronic neural damage. Secondly, the trained immunity phenotype induced by individual cytokines here provides a candidate research model that mimics the pathogenesis of human neuroinflammatory diseases with chronic duration. The training effect on microglia lasts for a long time, even after the original stimuli vanished. Accordingly, our data identified the training effect of BAFF on microglia last one month after the last ICV administration of BAFF (**Supplementary Figure 9**). Since BAFF expression in patients’ CSF was closely associated with the relapse of MS or BD (43, 44), it provides a possibility that BAFF may exert a signature on microglial cells during or even before the onset of the diseases. With the progression of the neurological diseases, another pulse of BAFF penetrates to the brain through the in-completed blood-brain barrier or released from brain-resident astrocytes (45) and then restimulates the BAFF-priming microglial cells to elicit a rapid and robust inflammatory response. Taken together, trained immunity may be a novel mechanism underlying the pathogenesis of human neuroinflammatory diseases with remission and relapse. Microglia would be the missing link by remembering peripheral inflammation and years later accelerating the outbreak of autoimmune or inflammatory neuro diseases. Finally, pharmacological blockage of mTOR by rapamycin mounts a protective effect on the trained immunity of microglia, indicating a potential therapeutic role of rapamycin in neuroimmunological diseases in which BAFF is involved.

In conclusion, we provide the first in-vitro and in-vivo evidence that BAFF exposure induces trained immunity-like phenotype of microglial cells accompanied by long-lasting enhanced glycolysis and Akt/mTOR/HIF-1α signaling pathway activation. Besides, our current data highlights the potential of rapamycin-based therapies for the treatment of neuroimmunological diseases.

## Data Availability Statement

The original contributions presented in the study are included in the article/**Supplementary Material**. Further inquiries can be directed to the corresponding author.

## Ethics Statement

The animal study was reviewed and approved by the animal care and use committee of First Affiliated Hospital of China Medical University.

## Author Contributions

JNW carried out most of the experiments. CSY participated in the immunoassays and helped to edit the manuscript. XYH, JYX, and YY participated in the culture of mouse primary microglial cells. LQ conceived of the study, and participated in its design and coordination. PTY designed and coordinated the study, and wrote the manuscript. All authors contributed to the article and approved the submitted version.

## Funding

This work was supported by the following grants: foundation from the Project for Construction of Key Platform, Shenyang, China (19-109-4-15 to PTY); foundation from Clinical Research Center for Immune diseases of Shenyang, Liaoning, China (20-204-4-43 and 18_009-4-03 to PTY); the Program of the Distinguished Professor of Liaoning Province (28020 to PTY); Doctoral Fund of Liaoning Province (no. 2019JH3/10100149 to JNW); and Science Foundation for the Youth Scholars of China Medical University (no. 1210519018 to JNW).

## Conflict of Interest

The authors declare that the research was conducted in the absence of any commercial or financial relationships that could be construed as a potential conflict of interest.

## Supplementary Material

Supplementary Figure 1 The effect of BAFF training on 1 cell viability and cell proliferation of BV2 cells. BV2 cells were treated as Figure 1A. On day 6, BV2 cells were restimulated with LPS, 24 h later, the cell viability **(A)** and proliferation **(B)** of BV2 cells were assayed by MTT and trypan blue assay, respectively(n=15). On day 6, BV2 cells were restimulated with BAFF, the cell viability **(C)** and proliferation **(D)** of BV2 cells were assayed on day 7(n=15). Data are means±SED; Kruskal-Wallis test with Turkey post hoc test; *p < 0.05 compared with CTL.

Supplementary Figure 2 The direct effect of BAFF priming on proinflammatory cytokines secretion in BV2 cells. BV2 cells were primed with different doses of BAFF(2.5ng/ml, 5ng/ml, 10ng/ml) or vehicle for 24 h. The expression of TNF-α **(A)**, IL-6 **(B)** and IL-1β **(C)** in the supernatants(n=12) were assayed by ELISA. Data are means±SED; Kruskal-Wallis test with Turkey post hoc test; *p < 0.05 compared with CTL.

Supplementary Figure 3 mRNA expression of BAFF receptors in BV2 cells BV2 cells were primed with 10ng/ml BAFF or vehicle for 24 h. On day 6, the relative mRNA expression of TACI, BAFF-R, and BCMA, relative to GAPDH, were assessed in BV2 cells by quantitative real-time PCR(qPCR). Data are means±SED (n=6); t-test; *p < 0.05.

Supplementary Figure 4 Down-regulation of TACI by siRNA. Control siRNA (NC-siRNA) or TACI siRNA(TACI-siRNA) was transfected into BV2 cells, 16h later RNA was subjected to qPCR. GAPDH represents the loading control. Data are mean ± SEM (n=6); Kruskal-Wallis test with Turkey post hoc test; **p < 0.01.

Supplementary Figure 5 Effects of TACI siRNA on BAFF training of BV2 cells. BV2 cells were transfected with TACI siRNA(TACI-siRNA) or control siRNA(NC-siRNA) for 16h and then primed with vehicle or BAFF for 24 h. After a rested of 5 days, cells were restimulated with LPS or BAFF for 24 h. The amounts of TNF-α **(A)** and IL-6 **(B)** were measured in the culture supernatants and the cytotoxicity of transfection was measured by MTT **(C)** on day 7 upon LPS restimulation (n=12). The secretion of TNF-α **(D)** and IL-6 **(E)** and the cytotoxicity **(F)** were measured on day 7 upon BAFF restimulation (n=12). The Data are mean ± SEM (n=12); Kruskal-Wallis test with Turkey post hoc test; *p < 0.05, **p < 0.01, and ***p < 0.001.

Supplementary Figure 6 Inhibition of Akt or HIF-1α suppressed BAFF training of BV2 cells upon LPS restimulation. (A-C) BV2 cells were pretreated with different doses of MK2206(50nM, 100nM, 200nM), and then primed with BAFF for 24 h. After a rested of 5 days, cells were restimulated with LPS for 24 h. The amounts of TNF-α **(A)** and IL-6 **(B)** were measured in the culture supernatants and the cytotoxicity of MK2206 was measured by MTT **(C)** on day 7(n=12). (D-F) BV2 cells were pretreated with different doses of 2-ME(0.5μM, 1μM, 2μM), and then primed with BAFF for 24 h. The secretion of TNF-α **(D)** and IL-6 **(E)** and the cytotoxicity of 2-ME **(F)** were measured on day 7 upon LPS restimulation (n=12). The Data are mean ±SEM; Kruskal-Wallis test with Turkey post hoc test; *p < 0.05, **p < 0.01, and ***p < 0.001.

Supplementary Figure 7 mRNA expression of BAFF receptors in primary microglial Cells. Primary microglial cells were primed with 10ng/ml BAFF or vehicle for 24 h. On day1 **(A)** and day 6 **(B)**, the relative mRNA expression of TACI, BAFF-R, and BCMA, relative to GAPDH, were assessed by quantitative real-time PCR(qPCR). Data are means ± SED (n=6); t-test; *p < 0.05.

Supplementary Figure 8 BAFF training induced CCL2 production in primary microglial cells. Primary microglia were pretreated with 100nM rapamycin for 1 h and then treated with BAFF(10ng/ml) or vehicle for 24 h. On day 6, microglial cells were restimulated with BAFF(40ng/ml) for 24 h. The amount of CCL2 was measured in the culture supernatants on day 7.Data are mean ± SEM (n = 9); Kruskal-Wallis test with Turkey post hoc test;*p < 0.05, **p < 0.01, and ***p < 0.001.

Supplementary Figure 9 Immunofluorescence staining of Iba1 in the cortex and hippocampus. Mice were trained with BAFF or vehicle. One month later, the brain section was stained with Iba1 antibody to evaluate the number and morphological change of microglia in the cortex **(A)** and hippocampus **(C)**. Comparisons of the numbers of Iba1-positive microglia in the cortex **(B)** and hippocampus **(D)**. A representative Iba-1+ cell from the cortex is shown magnified in the red box in the right upper corner of the image. Data are mean ±SEM(n = 6); t-test; *p < 0.05.

## Abbreviations

BAFF, B cell-activating factor; oxLDL, oxidized low-density lipoprotein; LXR, liver X receptor; CNS, central nervous system; LPS, lipopolysaccharide; TNF, tumor necrosis factor; IL, interleukin; CCL, C-C motif ligand; TACI, transmembrane activator and calcium modulator and cyclophilin ligand interactor; BCMA, B-cell maturation antigen; BAFFR, BAFF receptor; AD, Alzheimer’s disease; CSF, cerebral spinal fluid; MS, multiple sclerosis; NPSLE, neuropsychiatric systemic lupus erythematosus; BD, Behcet’s disease; mTOR, mammalian target of rapamycin; HIF-1α, hypoxia-inducible factor 1 alpha; ELISA, enzyme-linked immunosorbent assay; NAD+, nicotinamide adenine dinucleotide; NADH, reduced form of NAD+; OXPHOS, oxidative phosphorylation; NF-κB, nuclear factor kappa-light-chain-enhancer of activated B cells; TCA, glycolysis or tricarboxylic acid; siRNA, small interfering RNA; ChIP, chromatin Immunoprecipitation; ICV, intracerebroventricular injection; YMT, Y-maze test; NORT, novel object recognition test; H3K4me3, lysine 4 of histone H3; Iba1, ionized calcium-binding adaptor molecule 1.
