# Supplementary figures and images for: Rapamycin Modulates the Proinflammatory Memory-Like Response of Microglia Induced by BAFF
Source: Front Immunol. 2021 May 12;12:639049. doi: 10.3389/fimmu.2021.639049 (PMC8158300; doi:10.3389/fimmu.2021.639049)

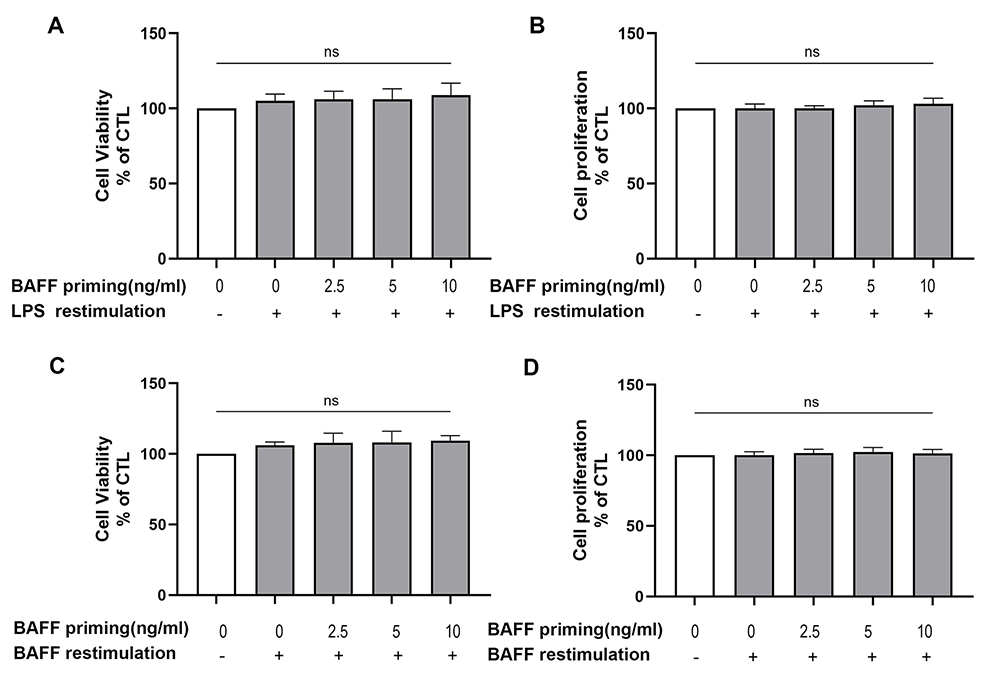

Supplement: Supplementary Figure 1 — The effect of BAFF training on 1 cell viability and cell proliferation of BV2 cells. BV2 cells were treated as Figure 1A. On day 6, BV2 cells were restimulated with LPS, 24 h later, the cell viability (A) and proliferation (B) of BV2 cells were assayed by MTT and trypan blue assay, respectively(n=15). On day 6, BV2 cells were restimulated with BAFF, the cell viability (C) and proliferation (D) of BV2 cells were assayed on day 7(n=15). Data are means±SED; Kruskal-Wallis test with Turkey post hoc test; *p < 0.05 compared with CTL. [file Image_1.tif]

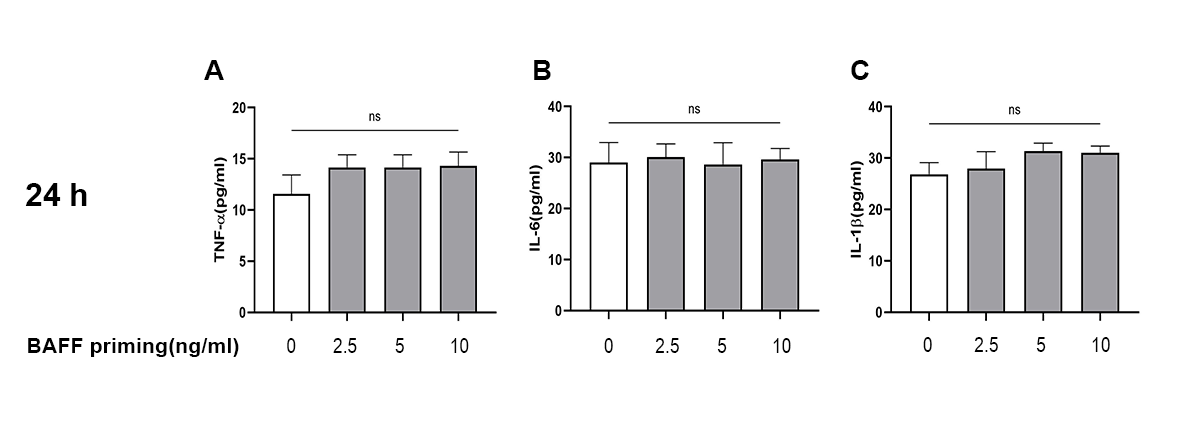

Supplement: Supplementary Figure 2 — The direct effect of BAFF priming on proinflammatory cytokines secretion in BV2 cells. BV2 cells were primed with different doses of BAFF(2.5ng/ml, 5ng/ml, 10ng/ml) or vehicle for 24 h. The expression of TNF-α (A), IL-6 (B) and IL-1β (C) in the supernatants(n=12) were assayed by ELISA. Data are means±SED; Kruskal-Wallis test with Turkey post hoc test; *p < 0.05 compared with CTL. [file Image_2.tif]

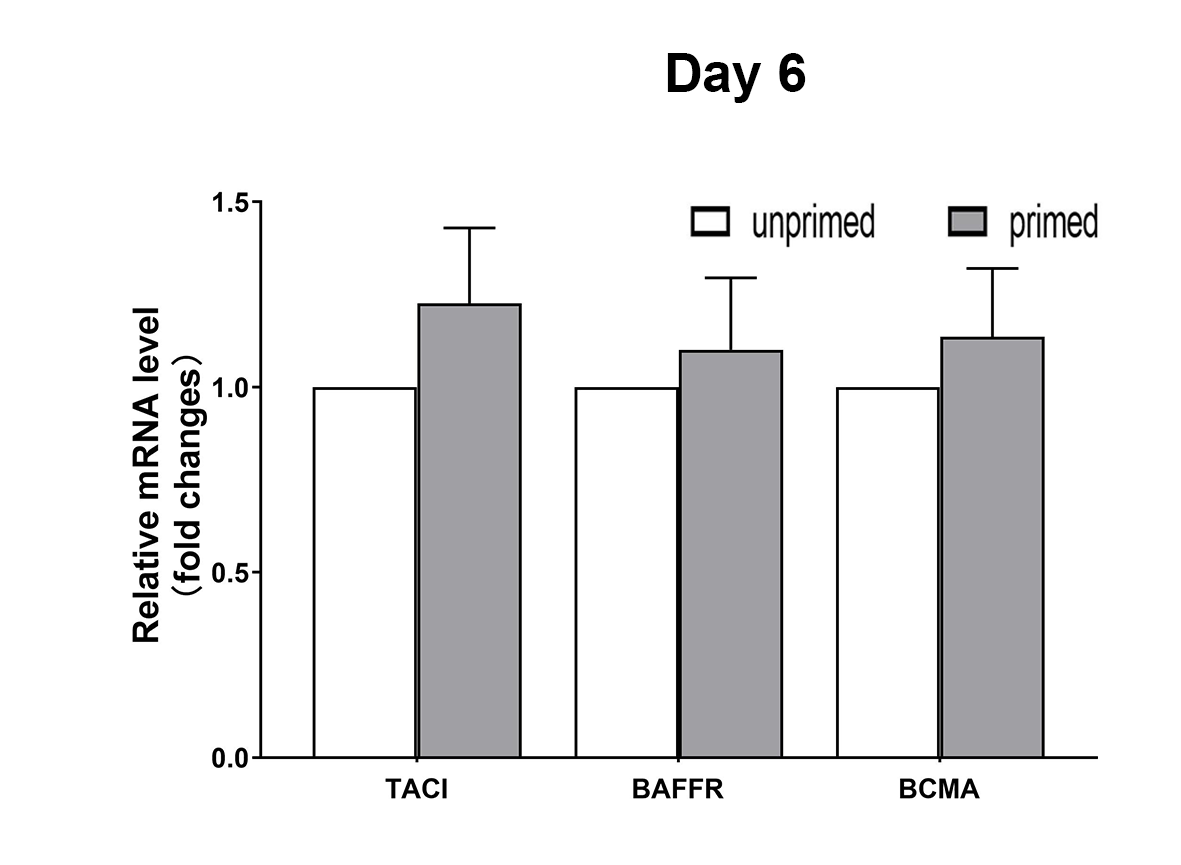

Supplement: Supplementary Figure 3 — mRNA expression of BAFF receptors in BV2 cells BV2 cells were primed with 10ng/ml BAFF or vehicle for 24 h. On day 6, the relative mRNA expression of TACI, BAFF-R, and BCMA, relative to GAPDH, were assessed in BV2 cells by quantitative real-time PCR(qPCR). Data are means±SED (n=6); t-test; *p < 0.05. [file Image_3.tif]

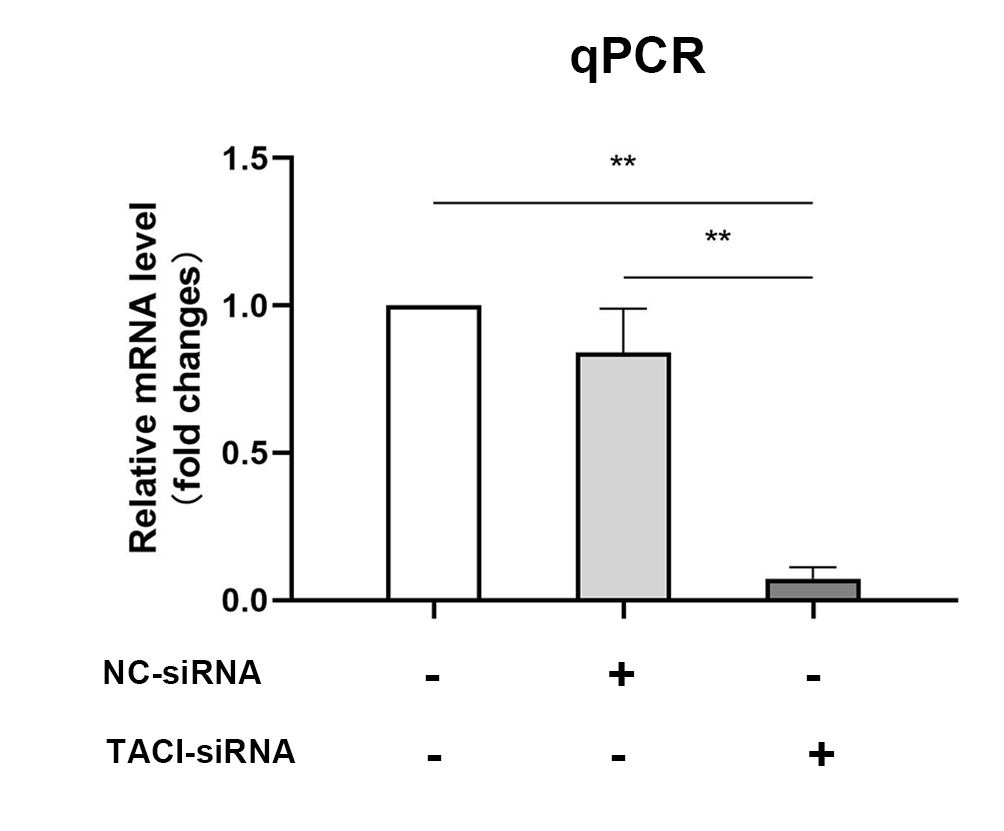

Supplement: Supplementary Figure 4 — Down-regulation of TACI by siRNA. Control siRNA (NC-siRNA) or TACI siRNA(TACI-siRNA) was transfected into BV2 cells, 16h later RNA was subjected to qPCR. GAPDH represents the loading control. Data are mean ± SEM (n=6); Kruskal-Wallis test with Turkey post hoc test; **p < 0.01. [file Image_4.tif]

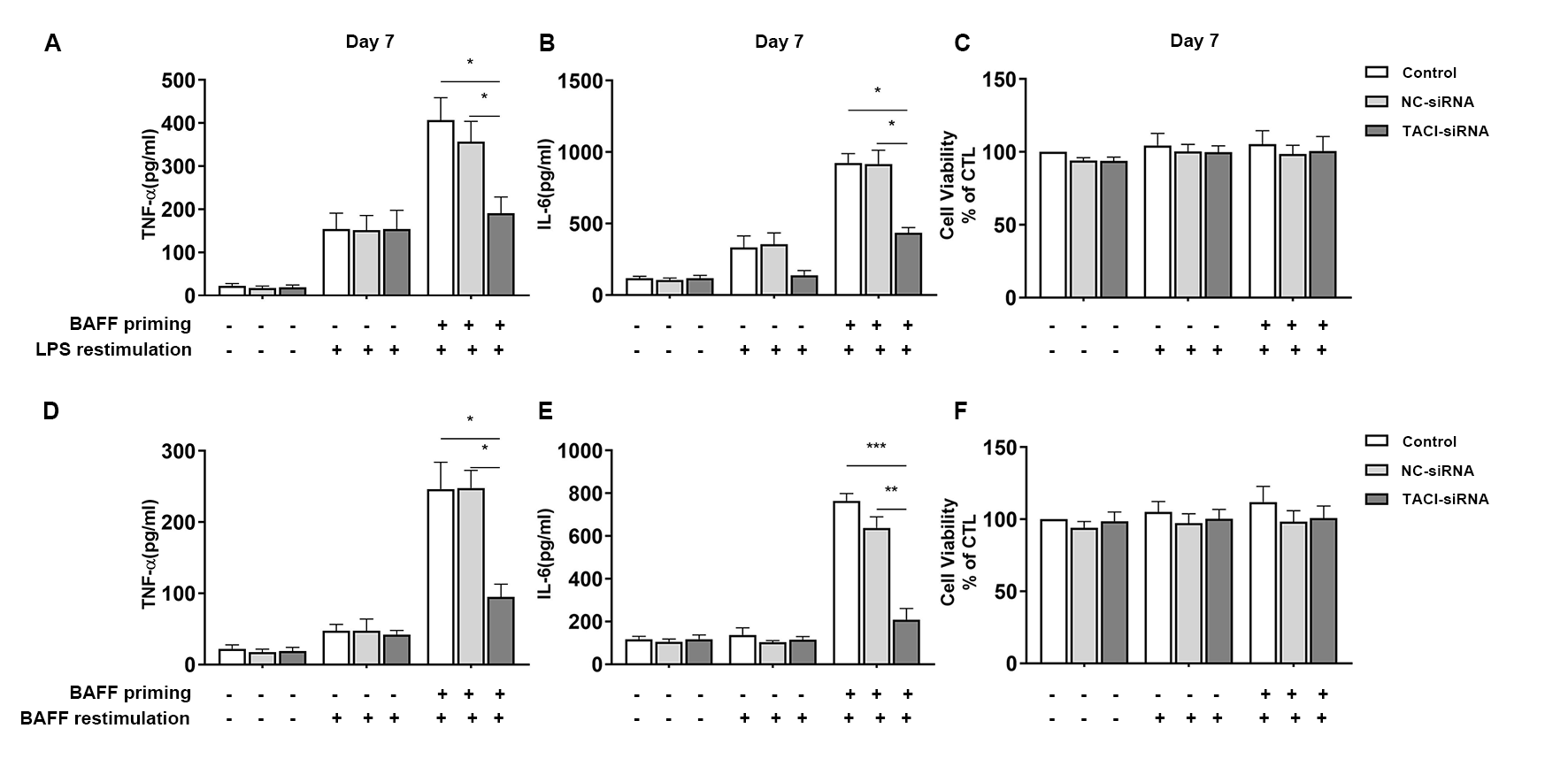

Supplement: Supplementary Figure 5 — Effects of TACI siRNA on BAFF training of BV2 cells. BV2 cells were transfected with TACI siRNA(TACI-siRNA) or control siRNA(NC-siRNA) for 16h and then primed with vehicle or BAFF for 24 h. After a rested of 5 days, cells were restimulated with LPS or BAFF for 24 h. The amounts of TNF-α (A) and IL-6 (B) were measured in the culture supernatants and the cytotoxicity of transfection was measured by MTT (C) on day 7 upon LPS restimulation (n=12). The secretion of TNF-α (D) and IL-6 (E) and the cytotoxicity (F) were measured on day 7 upon BAFF restimulation (n=12). The Data are mean ± SEM (n=12); Kruskal-Wallis test with Turkey post hoc test; *p < 0.05, **p < 0.01, and ***p < 0.001. [file Image_5.tif]

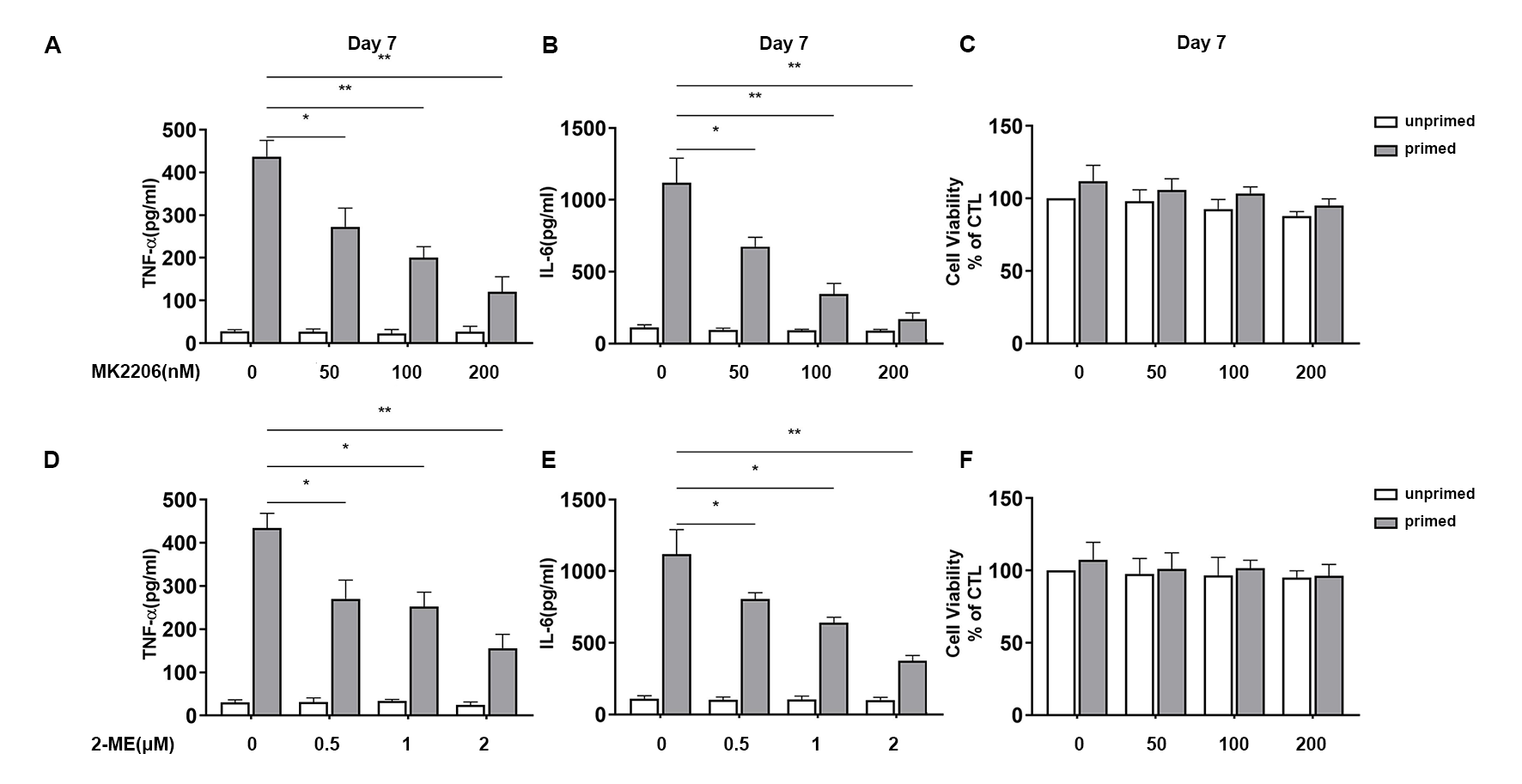

Supplement: Supplementary Figure 6 — Inhibition of Akt or HIF-1α suppressed BAFF training of BV2 cells upon LPS restimulation. (A-C) BV2 cells were pretreated with different doses of MK2206(50nM, 100nM, 200nM), and then primed with BAFF for 24 h. After a rested of 5 days, cells were restimulated with LPS for 24 h. The amounts of TNF-α (A) and IL-6 (B) were measured in the culture supernatants and the cytotoxicity of MK2206 was measured by MTT (C) on day 7(n=12). (D-F) BV2 cells were pretreated with different doses of 2-ME(0.5μM, 1μM, 2μM), and then primed with BAFF for 24 h. The secretion of TNF-α (D) and IL-6 (E) and the cytotoxicity of 2-ME (F) were measured on day 7 upon LPS restimulation (n=12). The Data are mean ±SEM; Kruskal-Wallis test with Turkey post hoc test; *p < 0.05, **p < 0.01, and ***p < 0.001. [file Image_6.tif]

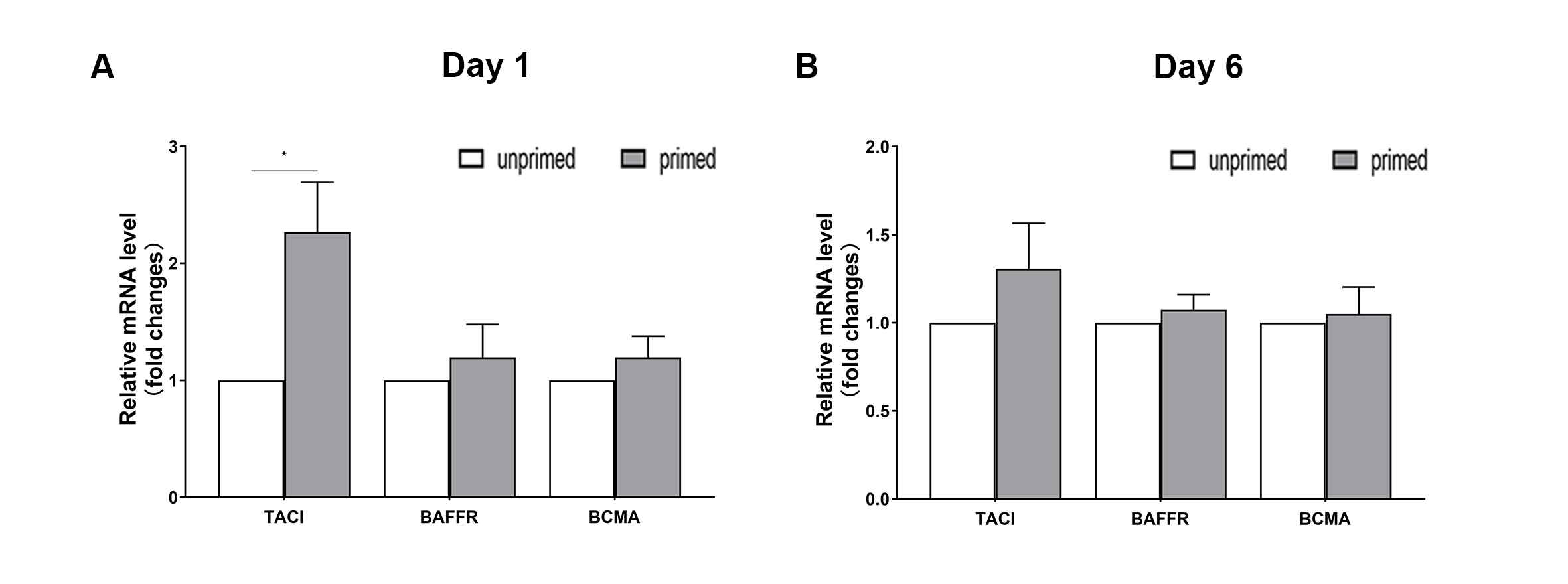

Supplement: Supplementary Figure 7 — mRNA expression of BAFF receptors in primary microglial Cells. Primary microglial cells were primed with 10ng/ml BAFF or vehicle for 24 h. On day1 (A) and day 6 (B), the relative mRNA expression of TACI, BAFF-R, and BCMA, relative to GAPDH, were assessed by quantitative real-time PCR(qPCR). Data are means ± SED (n=6); t-test; *p < 0.05. [file Image_7.tif]

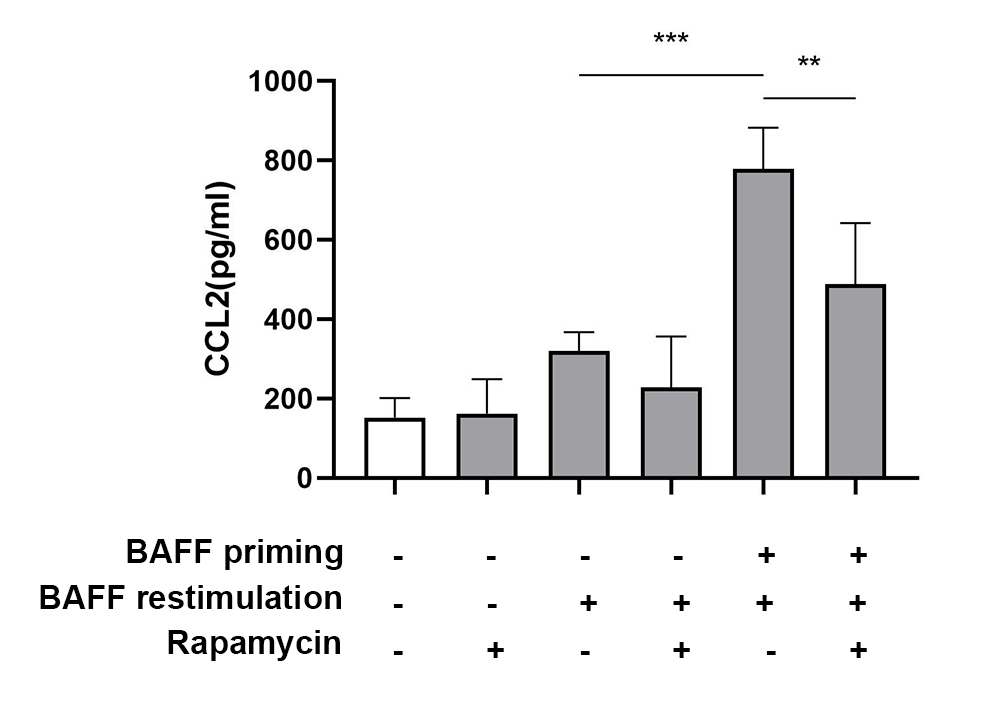

Supplement: Supplementary Figure 8 — BAFF training induced CCL2 production in primary microglial cells. Primary microglia were pretreated with 100nM rapamycin for 1 h and then treated with BAFF(10ng/ml) or vehicle for 24 h. On day 6, microglial cells were restimulated with BAFF(40ng/ml) for 24 h. The amount of CCL2 was measured in the culture supernatants on day 7.Data are mean ± SEM (n = 9); Kruskal-Wallis test with Turkey post hoc test;*p < 0.05, **p < 0.01, and ***p < 0.001. [file Image_8.jpeg]

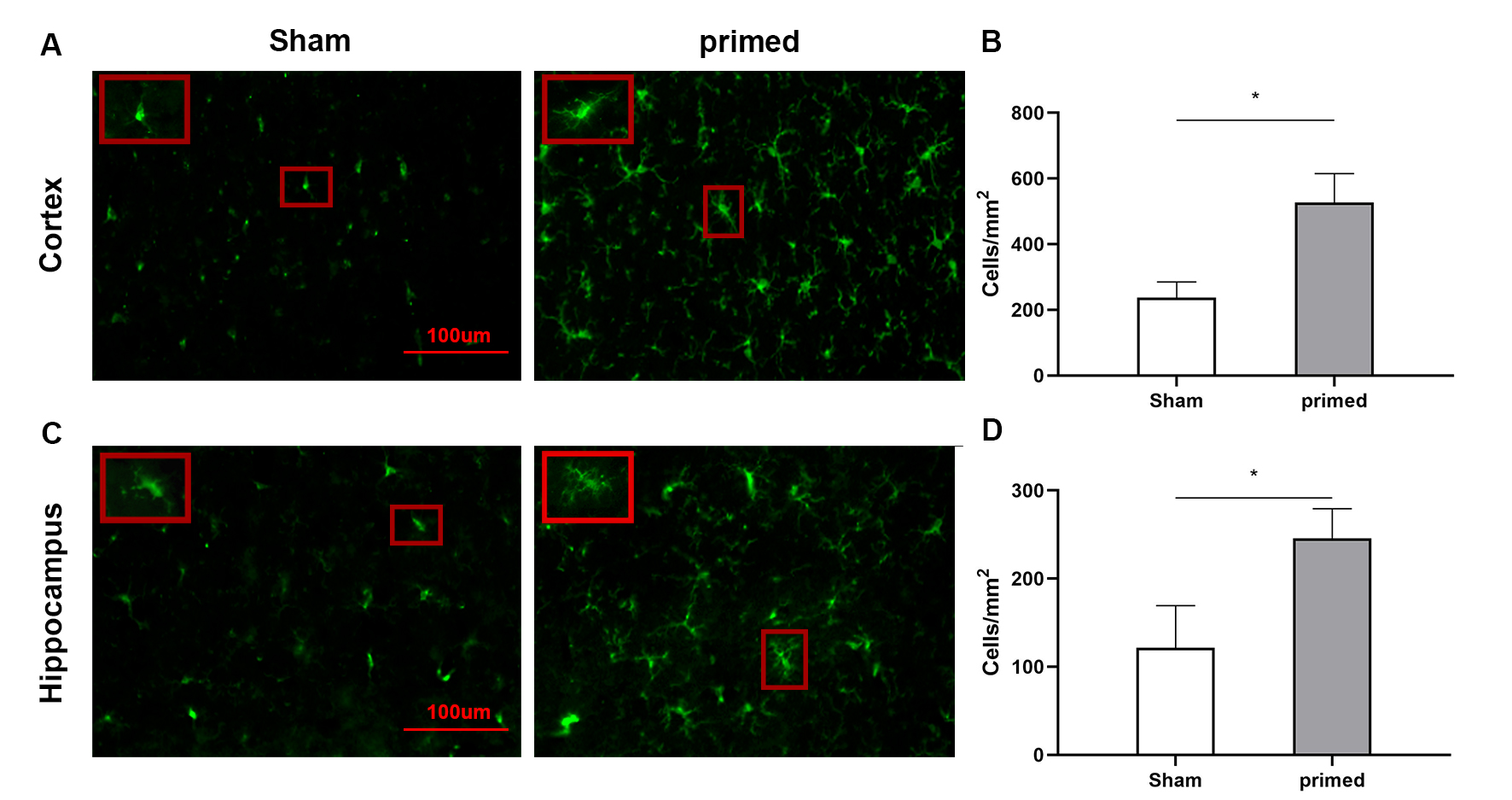

Supplement: Supplementary Figure 9 — Immunofluorescence staining of Iba1 in the cortex and hippocampus. Mice were trained with BAFF or vehicle. One month later, the brain section was stained with Iba1 antibody to evaluate the number and morphological change of microglia in the cortex (A) and hippocampus (C). Comparisons of the numbers of Iba1-positive microglia in the cortex (B) and hippocampus (D). A representative Iba-1+ cell from the cortex is shown magnified in the red box in the right upper corner of the image. Data are mean ±SEM(n = 6); t-test; *p < 0.05. [file Image_9.jpeg]
